# Circulating tryptophan–kynurenine pathway metabolites are associated with all‐cause mortality among patients with stage I–III colorectal cancer

**DOI:** 10.1002/ijc.35183

**Published:** 2024-09-23

**Authors:** Victoria Damerell, Niels Klaassen‐Dekker, Stefanie Brezina, Jennifer Ose, Arve Ulvik, Eline H. van Roekel, Andreana N. Holowatyj, Andreas Baierl, Jürgen Böhm, Martijn J. L. Bours, Hermann Brenner, Johannes H. W. de Wilt, William M. Grady, Nina Habermann, Michael Hoffmeister, Pekka Keski‐Rahkonen, Tengda Lin, Peter Schirmacher, Petra Schrotz‐King, Alexis B. Ulrich, Fränzel J. B. van Duijnhoven, Christy A. Warby, David Shibata, Adetunji T. Toriola, Jane C. Figueiredo, Erin M. Siegel, Christopher I. Li, Andrea Gsur, Ellen Kampman, Martin Schneider, Per M. Ueland, Matty P. Weijenberg, Cornelia M. Ulrich, Dieuwertje E. Kok, Biljana Gigic, FOCUS Consortium

**Affiliations:** ^1^ Department of General, Visceral and Transplantation Surgery Heidelberg University Hospital Heidelberg Germany; ^2^ Division of Human Nutrition and Health Wageningen University & Research Wageningen The Netherlands; ^3^ Center for Cancer Research Medical University of Vienna Vienna Austria; ^4^ Huntsman Cancer Institute Salt Lake City Utah USA; ^5^ Department of Population Health Sciences University of Utah Salt Lake City Utah USA; ^6^ Department III: Media, Information and Design University of Applied Sciences and Arts, Hochschule Hannover Hannover Germany; ^7^ BEVITAL Bergen Norway; ^8^ Department of Epidemiology, GROW School for Oncology and Reproduction Maastricht University Maastricht The Netherlands; ^9^ Department of Medicine Vanderbilt University Medical Center Nashville Tennessee USA; ^10^ Department of Statistics and Operations Research University of Vienna Vienna Austria; ^11^ Division of Preventive Oncology National Center for Tumor Diseases and German Cancer Research Center Heidelberg Germany; ^12^ Division of Clinical Epidemiology and Aging Research German Cancer Research Center (DKFZ) Heidelberg Germany; ^13^ German Cancer Consortium (DKTK) German Cancer Research Center (DKFZ) Heidelberg Germany; ^14^ Department of Surgery, Division of Surgical Oncology and Gastrointestinal Surgery Radboud University Medical Center Nijmegen The Netherlands; ^15^ Therapeutics and Translational Science Division Fred Hutchinson Cancer Research Center Seattle Washington USA; ^16^ Genome Biology European Molecular Biology Laboratory (EMBL) Heidelberg Germany; ^17^ Nutrition and Metabolism Branch International Agency for Research on Cancer Lyon France; ^18^ Institute of Pathology University of Heidelberg Heidelberg Germany; ^19^ Rheinland Klinikum Neuss Lukas Krankenhaus Neuss Germany; ^20^ Department of Surgery University of Tennessee Health Science Center Memphis Tennessee USA; ^21^ Department of Surgery Washington University St. Louis St. Louis Missouri USA; ^22^ Department of Medicine, Samuel Oschin Comprehensive Cancer Institute Cedars‐Sinai Medical Center California Los Angeles USA; ^23^ Department of Cancer Epidemiology H. Lee Moffitt Cancer Center and Research Institute Tampa Florida USA; ^24^ Division of Public Health Sciences Fred Hutchinson Cancer Center Seattle Washington USA

**Keywords:** all‐cause mortality, colorectal cancer, kynurenines, prognosis, tryptophan

## Abstract

Alterations within the tryptophan–kynurenine metabolic pathway have been linked to the etiology of colorectal cancer (CRC), but the relevance of this pathway for prognostic outcomes in CRC patients needs further elucidation. Therefore, we investigated associations between circulating concentrations of tryptophan–kynurenine pathway metabolites and all‐cause mortality among CRC patients. This study utilizes data from 2102 stage I–III CRC patients participating in six prospective cohorts involved in the international FOCUS Consortium. Preoperative circulating concentrations of tryptophan, kynurenine, kynurenic acid (KA), 3‐hydroxykynurenine (HK), xanthurenic acid (XA), 3‐hydroxyanthranilic acid (HAA), anthranilic acid (AA), picolinic acid (PA), and quinolinic acid (QA) were measured by liquid chromatography–tandem mass spectrometry. Using Cox proportional hazards regression, we examined associations of above‐mentioned metabolites with all‐cause mortality, adjusted for potential confounders. During a median follow‐up of 3.2 years (interquartile range: 2.2–4.9), 290 patients (13.8%) deceased. Higher blood concentrations of tryptophan, XA, and PA were associated with a lower risk of all‐cause mortality (per doubling in concentrations: tryptophan: HR = 0.56; 95%CI:0.41,0.76, XA: HR = 0.74; 95%CI:0.64,0.85, PA: HR = 0.76; 95%CI:0.64,0.92), while higher concentrations of HK and QA were associated with an increased risk of death (per doubling in concentrations: HK: HR = 1.80; 95%CI:1.47,2.21, QA: HR = 1.31; 95%CI:1.05,1.63). A higher kynurenine‐to‐tryptophan ratio, a marker of cell‐mediated immune activation, was associated with an increased risk of death (per doubling: HR = 2.07; 95%CI:1.52,2.83). In conclusion, tryptophan–kynurenine pathway metabolites may be prognostic markers of survival in CRC patients.

## What's new?

Tryptophan is catabolized by the kynurenine pathway, which generates several different metabolites. Improper activation of this pathway may play a role in colorectal cancer (CRC). Here, the authors show for the first time that in CRC patients, an increased kynurenine‐to‐tryptophan ratio prior to surgery is associated with an increased risk of death. They also found that higher concentrations of certain metabolites (tryptophan, xanthurenic acid, and picolinic acid) were associated with lower mortality, while others (3‐hydroxykynurenine and quinolinic acid) were associated with higher mortality over a follow up period of 3 years.

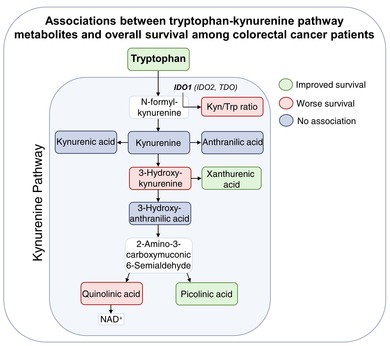

## 1 INTRODUCTION

Colorectal cancer (CRC) is second most common cause of cancer‐related death worldwide, with an estimated 1.9 million new cases and almost 1 million deaths in 2020.^1^ Several studies have shown that aberrant activation of the tryptophan–kynurenine pathway is linked to the etiology of CRC and other chronic diseases such as obesity, type 2 diabetes, and cardiovascular disease (CVD).^2^, ^3^, ^4^, ^5^, ^6^, ^7^ Tryptophan is an essential amino acid that is important for protein synthesis and it is also a precursor for metabolites involved in numerous (patho)physiological processes related to the nervous, endocrine, and immune system.^7^ Tryptophan is catabolized through three pathways: (1) the kynurenine pathway, leading to kynurenine and its downstream products via tryptophan 2,3‐dioxygenase (TDO; hepatic) and indoleamine 2,3‐dioxygenase (IDO; extrahepatic); (2) the serotonin pathway, leading to serotonin and its metabolites through tryptophan hydroxylase 1; and (3) direct metabolism by the gut microbiota into indole derivatives.^7^

**FIGURE 1:**
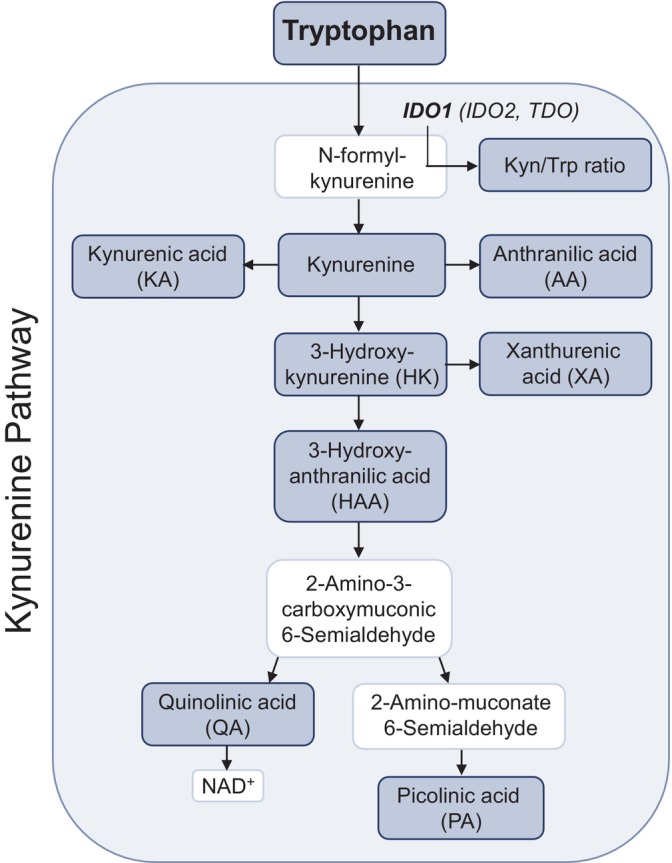
Simplified schematic representation of the tryptophan–kynurenine pathway. Kynurenine pathway metabolites addressed in this study are highlighted in dark blue.

Over 95% of tryptophan is catabolized via the kynurenine pathway which generates several downstream metabolites including kynurenine, kynurenic acid (KA), anthranilic acid (AA), 3‐hydroxykynurenine (HK), xanthurenic acid (XA), 3‐hydroxyanthranilic acid (HAA), picolinic acid (PA), and quinolinic acid (QA) (Figure 1).^2^ Preclinical research has shown that some of these metabolites may have anti‐inflammatory, anti‐oxidative, and neuroprotective properties (i.e., KA and PA), whereas others may have pro‐inflammatory, pro‐oxidative, and neurotoxic properties (i.e., HK and QA).^8^, ^9^ IDO1 is the most well studied enzyme that initiates tryptophan's catabolism to kynurenine in almost all major organs, except the liver,^10^ and its activity can be indirectly estimated by the kynurenine‐to‐tryptophan (Kyn/Trp) ratio.^10^ Since the activity of IDO1 is modulated by cytokines, in particular interferon‐γ (IFN‐γ), which are released upon inflammation and immune cell activation, the Kyn/Trp ratio is considered a well‐established marker of cell‐mediated immune activation.^10^, ^11^ Experimental research has shown that IDO1 promotes an immunosuppressive tumor microenvironment by inactivating T cells and natural killer cells as well by activating regulatory T cells, myeloid‐derived suppressor cells, and dendritic cells.^12^, ^13^, ^14^ Furthermore, IDO1 can promote CRC cell proliferation and inhibit apoptosis via the activation of the PI3K‐Akt signaling pathway.^15^

Studies revealed that CRC patients have reduced tryptophan levels and elevated levels of kynurenine pathway metabolites, indicating increased IDO1 activity, as compared to cancer‐free controls.^16^, ^17^ We have previously shown that higher plasma tryptophan levels were associated with lower risk of colon cancer and that a higher Kyn/Trp ratio was associated with higher colon cancer risk in a cohort of 110 patients and 153 controls.^18^ However, to our knowledge, there are no prospective studies on the role of tryptophan–kynurenine pathway metabolites in relation to survival in CRC patients. This is the first study to investigate preoperative circulating tryptophan–kynurenine pathway metabolites in relation to all‐cause mortality in patients diagnosed with stage I–III CRC.

## 2 MATERIALS AND METHODS

### 2.1 Study population

**FIGURE 2:**
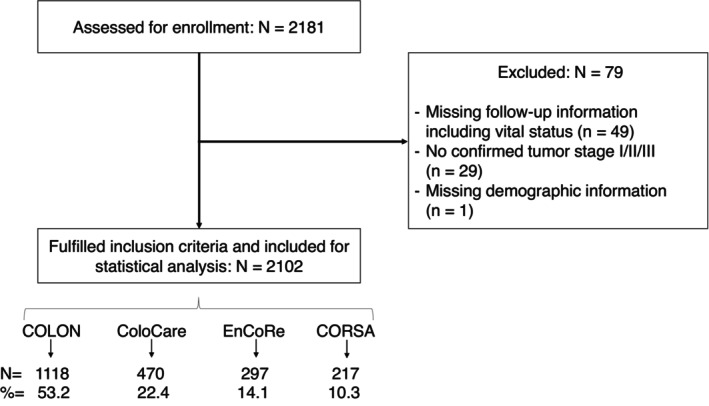
Study enrollment flow chart for the FOCUS Consortium.

This study utilizes data from 2102 non‐metastatic patients with stage I–III CRC who were recruited through six international cohorts participating in the FOCUS Consortium.^19^ The purpose of the FOCUS Consortium is to investigate associations between folate and folate‐mediated one‐carbon metabolism biomarkers and patient‐reported^20^ and clinical outcomes^21^ among patients diagnosed with primary CRC and aged 18 years or older.^19^ Briefly, the FOCUS Consortium comprises CRC patients from the ColoCare Study^22^ (study sites: Heidelberg University Hospital, Germany; Huntsman Cancer Institute, University of Utah, USA; and Fred Hutchinson Cancer Research Center, USA), the Colorectal Cancer Study of Austria (CORSA),^23^ the COLON (COlorectal cancer: Longitudinal, Observational study on Nutritional and lifestyle factors that may influence colorectal tumor recurrence, survival and quality of life) Study,^24^ and the Energy for life after ColoRectal cancer (EnCoRe) Study,^25^ both from the Netherlands. We collected detailed data on clinical and sociodemographic features, including patient age at diagnosis, sex, tumor stage, tumor site, (neo)adjuvant treatment, and data on all‐cause mortality. Lifestyle and anthropometric data including body mass index (BMI [kg/m^2^]), adherence to physical activity guidelines (self‐reported engagement in at least 150 min per week of moderate‐to‐vigorous physical activity), and smoking status (current, former, never) were collected at the time of diagnosis and harmonized across all study sites. Among the 2181 CRC patients within the FOCUS Consortium, 79 patients were excluded due to either no confirmed tumor stage I/II/III (*n* = 2 patients with stage 0, *n* = 27 patients with stage IV CRC [since this study only focuses on non‐metastatic CRC patients]), missing follow‐up information including vital status (*n* = 49), or missing demographic information (*n* = 1). The final cohort for analyses included 2102 patients diagnosed with stage I–III CRC (Figure 2).

### 2.2 Measurement of biomarkers of tryptophan–kynurenine pathway metabolism

Preoperative plasma (CORSA, COLON and EnCoRe) and serum (ColoCare Study) samples from CRC patients were processed as previously described.^19^ Circulating concentrations of tryptophan–kynurenine pathway metabolites (tryptophan, kynurenine, HK, KA, HAA, AA, XA, PA, and QA) and creatinine were analyzed by liquid chromatography‐mass spectrometry/mass spectrometry (LC–MS/MS) at BEVITAL AS (www.bevital.no).^26^ While the majority of patients (77.6%) were recruited at the time of diagnosis, prior to any cancer treatment, 22.4% of patients were recruited prior to surgery (i.e., neoadjuvant treatment might have occurred prior to baseline blood and data collection). Nevertheless, no blood draw occurred within 2 weeks of neoadjuvant chemotherapy, limiting the influence of ongoing treatments on blood biomarkers.^19^

### 2.3 Study endpoint

The primary endpoint of the present study was all‐cause mortality, defined as the time from pre‐surgery blood draw to the date of last follow up or death due to any cause. Data on vital status was collected from the clinical cancer registries or the inhabitant registries that exist at all study sites. For COLON and EnCoRe, all‐cause mortality data were collected from the Municipal Personal Records Database of the Netherlands. Survival data for CORSA participants were obtained by the Main Association of Austrian Social Insurance Carriers as well as from Statistics Austria.^19^ For ColoCare Study participants, the vital status or survival of patients was determined through local medical records, routine follow‐up mailings, periodic requests for external medical records, and state or national cancer and death registries. It is important to note that our dataset does not include specific causes of death. This limitation arises from the data collection process, where information on cause of death was not available for all cohorts. As a result, our analysis focuses on all‐cause mortality without distinguishing between cancer‐specific and other causes of mortality.

### 2.4 Statistical analysis

The characteristics of the study participants were summarized by presenting the frequency and percentage for categorical variables (e.g., sex, tumor site, tumor stage, (neo)adjuvant treatment) or mean and standard deviation (SD) for continuous variables (e.g., age, BMI) (Table 1). Statistical analyses were conducted using biomarkers of the tryptophan–kynurenine pathway metabolism, measured as continuous values as well as concentrations categorized in tertiles (tertiles were created by the overall cohort or by per cohort tertile assignment). The concentrations of tryptophan–kynurenine pathway metabolites and creatinine from all cohorts were merged and then log2‐transformed before analysis to approach normal distributions, thus, hazard ratios (HRs) should be interpreted per doubling in biomarker concentrations. The Kyn/Trp ratio was calculated by dividing the kynurenine by tryptophan (non‐transformed concentrations), log2‐transformed thereafter.

**TABLE 1:** Demographic, lifestyle, and primary tumor characteristics of the study population stratified by alive and deceased CRC patients: FOCUS Consortium.^a^

Characteristics	All	Alive	Deceased
Number of participants, *n*	2102	1812 (86.2)	290 (13.8)
Sex			
Female, *n* (%)	754 (35.9)	660 (36.4)	94 (32.4)
Male, *n* (%)	1348 (64.1)	1152 (63.6)	196 (67.6)
Age at diagnosis (years), mean (SD)	65.4 (10.2)	64.6 (10.1)	70.5 (10.0)
Body mass index (kg/m^2^), mean (SD)	27.1 (4.6)	27.1 (4.7)	27.3 (4.4)
Smoking status, *n* (%)			
Current	258 (12.3)	220 (12.1)	38 (13.1)
Former	1060 (50.4)	920 (50.8)	140 (48.3)
Never	672 (31.9)	583 (32.2)	89 (30.7)
Unknown/missing	112 (5.3)	89 (4.9)	23 (7.9)
Adherence to physical activity guidelines*, *n* (%)			
Yes	1135 (54.0)	1015 (56.0)	120 (41.4)
No	617 (29.4)	531 (29.3)	86 (29.6)
Unknown	350 (16.6)	266 (14.7)	84 (29.0)
Tumor stage, *n* (%) **			
I	558 (26.6)	510 (28.1)	48 (16.6)
II	605 (28.8)	529 (29.2)	76 (26.2)
III	848 (40.3)	703 (38.8)	145 (50.0)
Unknown/unspecified	91 (4.3)	70 (3.9)	21 (7.2)
Tumor site, *n* (%)			
Ascending colon	598 (28.4)	502 (27.7)	96 (33.1)
Descending colon	663 (31.5)	575 (31.7)	88 (30.3)
Rectosigmoid junction/rectum	803 (38.2)	701 (38.7)	102 (35.2)
Unknown	38 (1.8)	34 (1.9)	4 (1.4)
Surgical treatment, *n* (%)			
No	61 (2.9)	49 (2.7)	12 (4.1)
Yes	2006 (95.4)	1732 (95.6)	274 (94.5)
Unknown	35 (1.7)	31 (1.7)	4 (1.4)
Neoadjuvant treatment, *n* (%)			
No	1586 (75.5)	1372 (75.7)	214 (73.8)
Yes	470 (22.4)	400 (22.1)	70 (24.1)
Unknown	46 (2.1)	40 (2.2)	6 (2.1)
Type of neoadjuvant treatment, *n* (%)			
Chemotherapy	13 (2.8)	8 (2.0)	5 (7.1)
Radiation therapy	180 (38.3)	153 (38.2)	27 (38.6)
Chemoradiation	277 (58.9)	239 (59.8)	38 (54.3)
Unknown	0		
Adjuvant treatment, *n* (%)			
No	1204 (57.3)	1038 (57.3)	166 (57.2)
Yes	765 (36.3)	655 (36.1)	110 (37.9)
Unknown	133 (6.3)	119 (6.6)	14 (4.9)
Type of adjuvant treatment, *n* (%)			
Chemotherapy	556 (26.5)	473 (72.2)	83 (75.5)
Radiation therapy	170 (8.1)	150 (22.9)	20 (18.0)
Chemoradiation	31 (1.5)	25 (3.8)	6 (5.5)
Unknown	8 (0.4)	7 (1.1)	1 (1.0)
Cohort			
COLON	1118	977 (87.4)	141 (12.6)
EnCoRe	297	269 (90.6)	28 (9.4)
ColoCare FHCRC	131	107 (81.7)	24 (18.3)
ColoCare HCI	68	63 (92.6)	5 (7.4)
ColoCare HD	271	242 (89.3)	29 (10.7)
CORSA	217	154 (71.0)	63 (29.0)
Duration of follow‐up median (IQR) in years	3.2 (2.2–4.9)		
Cohort			
COLON	3.2 (2.4–4.7)	3.3 (2.5–4.9)	2.7 (1.0–4.0)
EnCoRe	2.9 (2.1–3.7)	3.0 (2.2–3.7)	1.0 (0.2–3.0)
ColoCare FHCRC	5.1 (4.7–5.4)	5.1 (5.0–5.4)	3.4 (2.4–4.8)
ColoCare HCI	2.2 (1.1–2.7)	2.2 (1.1–2.7)	0.4 (0.2–2.2)
ColoCare HD	2.1 (1.2–3.0)	2.1 (1.3–3.0)	1.5 (0.4–2.6)
CORSA	6.5 (4.8–11.4)	6.2 (4.7–9.9)	9.0 (5.2–13.9)
Biomarker concentration nmol/l: median (IQR)			
Tryptophan	61.2 (52.3–70.7)	61.7 (52.8–71.3)	57.9 (48.5–66.2)
Kynurenine	1.6 (1.4–1.9)	1.6 (1.4–1.9)	1.7 (1.5–2.1)
Kynurenic acid	47.7 (36.9–62.1)	47.6 (36.9–61.3)	49.0 (36.4–71.4)
Anthranilic acid	16.2 (12.8–21.6)	16.0 (12.8–21.0)	16.7 (12.7–23.9)
3‐Hydroxykynurenine	45.0 (35.0–58.4)	44.2 (34.4–56.5)	53.5 (38.8–77.3)
Xanthurenic acid	13.4 (8.8–18.8)	13.7 (9.0–19.1)	11.6 (6.7–17.2)
3‐Hydroxyanthranilic acid	40.2 (31.0–52.7)	40.2 (31.0–52.9)	40.5 (30.6–52.6)
Quinolinic acid	458 (361–603)	448 (356–584)	546 (406–754)
Picolinic acid	40.5 (28.6–55.9)	41.2 (29.0–56.1)	37.3 (26.7–53.8)
Kyn/Trp ratio	0.027 (0.022–0.032)	0.026 (0.022–0.031)	0.030 (0.025–0.038)

*Note*: *Self‐reported engagement in at least 150 min per week of moderate‐to‐vigorous physical activity. **Sixteen patients had a CRC that could not be distinguished between stage I–II or stage II–III disease.

Abbreviations: COLON, colorectal cancer: longitudinal, observational study on nutritional and lifestyle factors that may influence colorectal tumor recurrence, survival and quality of life Study; CORSA, Colorectal Cancer Study of Austria; EnCoRe, Energy for life after ColoRectal cancer; CRC, colorectal cancer; FHCRC, Fred Hutchinson Cancer Research Center; HCI, Huntsman Cancer Institute; HD, Heidelberg; IQR, interquartile range; Kyn/Trp ratio, kynurenine‐to‐tryptophan ratio; nmol: nanomolar; l, liter.

^a^ Values are presented as *n* (%) or mean and SD.

Chi‐square tests were applied to compare differences with regard to distribution of age, sex, tumor stage, tumor site, BMI, smoking history, and adherence to physical activity guidelines across biomarker tertiles for tryptophan, kynurenine and the Kyn/Trp ratio.

Adjusted HRs and 95% confidence intervals (CIs) were calculated using multivariable Cox proportional hazards regression analyses. Models were adjusted for potential confounders (as identified from literature^27^, ^28^, ^29^), including age at diagnosis (years), sex (men, women), tumor site (ascending colon: cecum to transverse colon, descending colon: splenic flexure to sigmoid colon, and rectosigmoid junction/rectum), tumor stage (I, II, III), and study cohort. Only complete datasets were considered for the multivariate analyses and numbers are indicated in the respective tables. Creatinine has been considered as covariate (to adjust for differences in clearance of tryptophan–kynurenine metabolites by the kidneys).

Since tryptophan metabolite levels were shown to differ between tumor stage and tumor site,^30^ we performed stratified analyses. Since the tryptophan–kynurenine pathway also plays a central role in obesity,^5^ we performed stratified analyses by BMI (normal weight (≥18.5 BMI <25), overweight (≥25 BMI <30), obese (≥30 BMI)). Further, stratified analysis was performed by study cohort. To exclude the possibility that changes in inflammatory burden related to BMI, smoking history or physical activity level or the administration of neoadjuvant or adjuvant treatment may impact biomarker concentrations,^27^, ^31^, ^32^, ^33^ we performed sensitivity analyses with additional adjustment for these covariates. Sensitivity analysis was performed by excluding participants who died within the first 60 days of blood draw.

Survival curves for overall survival by tryptophan metabolite tertiles (defined on the basis of the total study population) were calculated using Kaplan–Meier methods. The log‐rank test was used to compare survival between these groups. Heterogeneity in associations between biomarkers and all‐cause mortality stratified by tumor site and tumor stage was assessed using likelihood‐ratio tests for the comparison of the model fit for Cox proportional hazards regression models with and without corresponding interaction terms. All statistical analyses were conducted using SAS version 9.4 software. *p* values <0.05 were considered statistically significant.

## 3 RESULTS

### 3.1 Baseline characteristics

Table 1 presents the baseline characteristics of our study population stratified by alive and deceased CRC patients and Table S1 presents these characteristics stratified by study cohort. Table S2 additionally presents these characteristics stratified by tertiles of tryptophan, kynurenine and the Kyn/Trp ratio. Among the total study population, nearly two‐thirds were men and the average age at diagnosis was 65.4 years (SD: 10.2). Further, 67.3% of patients were from the Netherlands, 12.9% from Germany, 10.3% from Austria, and 9.4% from the United States. The mean BMI in this cohort was 27.1 kg/m^2^ (SD: 4.6), indicating that on average patients were classified as overweight. Current and former smokers accounted together for 63% of the study population (1318 out of 2102). In total, 40.3% of patients (848 out of 2102) were diagnosed with stage III CRC, and 38.2% (803 out of 2102) had a tumor localized in the rectosigmoid junction or rectum (rectal cancer). Neoadjuvant therapy was received by 22.4% of the patients, 95.4% underwent CRC resection, and 36.3% received adjuvant therapy.

### 3.2 Associations between circulating tryptophan–kynurenine pathway metabolites and CRC all‐cause mortality

**FIGURE 3:**
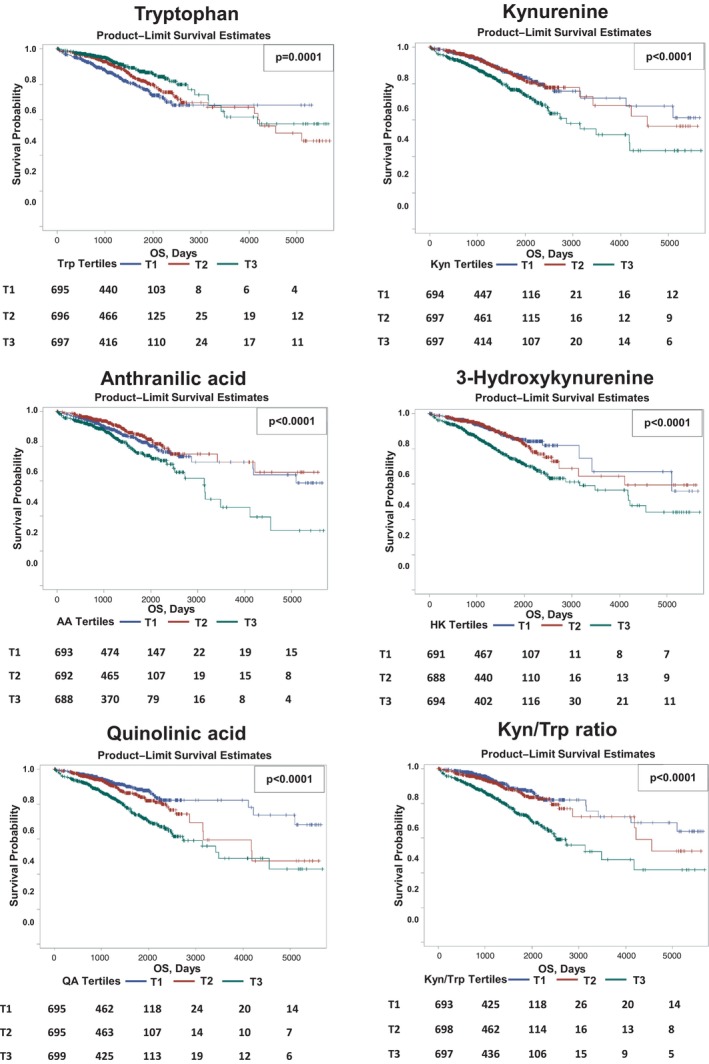
Selected Kaplan–Meier survival curves for unadjusted associations between tryptophan–kynurenine pathway metabolite concentrations with all‐cause mortality by tertiles among stage I–III colorectal cancer patients from the FOCUS Consortium. *p* value was calculated using log‐rank statistics. Tryptophan, T1: 10.8–55.4 nmol/L; T2: 55.4–67.3 nmol/L; T3: >67.3.1 nmol/L. Kyn, T1: 0.6–1.5 nmol/L; T2: 1.5–1.8 nmol/L; T3: >1.8 nmol/L. AA, T1: 3.4–13.9 nmol/L; T2: 13.9–19.3 nmol/L; T3: >19.3 nmol/L. HK, T1: 1.6–38.2 nmol/L; T2: 38.3–53.4 nmol/L; T3: >53.5 nmol/L. QA, T1: 74.2–390.0 nmol/L; T2: 391.0–544.0 nmol/L; T3: >545.0 nmol/L. Kyn/Trp ratio, T1: 0.012–0.024; T2: 0.024–0.030; T3: >0.030. AA, Anthranilic acid; FOCUS, folate‐dependent 1‐carbon metabolism in colorectal cancer recurrence and survival; HK, 3‐Hydroxykynurenine; Kyn, Kynurenine; QA, Quinolinic acid; T, tertile; Trp, Tryptophan.

Median follow‐up time was 3.2 years (interquartile range: 2.2–4.9 years). During follow‐up, 290 (13.8%) of the 2102 CRC patients deceased. Associations of tryptophan–kynurenine metabolites with all‐cause mortality are described in Table 2 (continuous scale and tertiles of the overall study cohort) and Kaplan–Meier survival curves are shown in Figure 3. A doubling in tryptophan concentration was associated with a 44% decreased risk of all‐cause mortality after adjustment (HR = 0.56; 95%CI:0.41,0.77). Comparing the top to the bottom tertile, we observed a 38% decrease in risk of all‐cause mortality for tryptophan (HR_T3‐T1_ = 0.62; 95%CI:0.45,0.85). Similarly, doublings in XA and PA concentrations were associated with a 26% and 24% decreased risk of all‐cause mortality, respectively (HR_XA_ = 0.74; 95%CI:0.64,0.85; HR_PA_ = 0.76; 95%CI:0.64,0.92) and a 41% and 33% decreased risk of death comparing the top to the bottom tertile, respectively (XA: HR_T3‐T1_ = 0.59; 95%CI:0.43,0.82; PA: HR_T3‐T1_ = 0.67; 95%CI:0.49,0.92). On the other hand, doublings in HK and QA concentrations were associated with a higher risk of death, for example, doubling of HK was associated with a 80% increase in risk of death (HR = 1.80; 95%CI:1.47,2.21), and a 43% increase in risk comparing the top to the bottom tertile (HR_T3‐T1_ = 1.43; 95%CI:1.01,2.01). A doubling in the Kyn/Trp ratio was associated with a 2.1‐fold increase in risk of death (HR = 2.07; 95%CI:1.52,2.83), and a 1.6‐fold increase in risk comparing the top to the bottom tertile (HR_T3‐T1_ = 1.64; 95%CI:1.16,2.32). Kynurenine, KA, AA, and HAA were not significantly associated with risk of death in the adjusted model. To exclude the possibility that the most robust estimates are associated with the largest cohort (in particular COLON), which comprises half of the overall study population, we additionally derived tertiles for each study cohort separately and repeated the analyses. Noteworthy, we observed similar results for all‐cause mortality (Table S3).

**TABLE 2:** Associations between tryptophan–kynurenine pathway metabolites and all‐cause mortality among patients with stage I–III CRC (*n* = 2102).

Biomarker	Median (IQR) concentration	All‐cause mortality	
		Crude HR (95% CI)	Adj. HR^a^ (95% CI)
Tryptophan			
Continuous^b^	61.2 (52.3–70.7)	**0.43 (0.32,0.58)**	**0.56 (0.41,0.77)**
T1	<55.4	Ref	Ref
T2	55.4–67.3	**0.74 (0.57,0.97)**	0.81 (0.61,1.08)
T3	>67.3	**0.53 (0.40,0.72)**	**0.62 (0.45,0.85)**
Kynurenine			
Continuous^b^	1.6 (1.4–1.9)	**2.37 (1.73,3.26)**	1.28 (0.86,1.92)
T1	<1.5	Ref	Ref
T2	1.5–1.8	1.03 (0.75,1.40)	0.87 (0.63,1.22)
T3	>1.8	**1.77 (1.33,2.34)**	1.07 (0.77,1.49)
Kyn/Trp ratio			
Continuous^b^	0.027 (0.022–0.032)	**3.09 (2.44,3.90)**	**2.07 (1.52,2.83)**
T1	<0.024	Ref	Ref
T2	0.024–0.030	1.25 (0.90,1.75)	1.11 (0.78,1.58)
T3	>0.030	**2.54 (1.89,3.42)**	**1.64 (1.16,2.32)**
Kynurenic acid (KA)			
Continuous^b^	47.7 (36.9–62.1)	**1.32 (1.10,1.58)**	0.87 (0.68,1.12)
T1	<40.8	Ref	Ref
T2	40.9–56.3	1.04 (0.78,1.40)	0.91 (0.66,1.25)
T3	>56.4	**1.41 (1.07,1.86)**	0.84 (0.66,1.19)
Anthranilic acid (AA)			
Continuous^b^	16.2 (12.8–21.6)	**1.38 (1.15,1.65)**	0.92 (0.72,1.17)
T1	<13.9	Ref	Ref
T2	13.9–19.3	0.79 (0.59,1.07)	**0.56 (0.40,0.77)**
T3	>19.3	**1.48 (1.13,1.94)**	0.83 (0.60,1.15)
3‐Hydroxykynurenine (HK)			
Continuous^b^	45.0 (35.0–58.4)	**2.10 (1.80,2.46)**	**1.80 (1.47,2.21)**
T1	<38.2	Ref	Ref
T2	38.3–53.4	1.09 (0.79,1.51)	0.98 (0.69,1.38)
T3	>53.5	**2.10 (1.57,2.80)**	**1.43 (1.01,2.01)**
Xanthurenic acid (XA)			
Continuous^b^	13.4 (8.8–18.8)	**0.82 (0.72,0.92)**	**0.74 (0.64,0.85)**
T1	<10.3	Ref	Ref
T2	10.3–16.8	**0.68 (0.52,0.90)**	**0.72 (0.53,0.97)**
T3	>16.9	**0.72 (0.54,0.95)**	**0.59 (0.43,0.82)**
3‐Hydroxyanthranilic acid (HAA)			
Continuous^b^	40.2 (31.0–52.7)	1.00 (0.83,1.21)	0.82 (0.66,1.03)
T1	<33.6	Ref	Ref
T2	33.7–47.8	0.92 (0.70,1.23)	0.80 (0.59,1.09)
T3	>47.9	1.03 (0.78,1.36)	0.80 (0.58,1.09)
Picolinic acid (PA)			
Continuous^b^	40.5 (28.6–55.9)	0.87 (0.75,1.03)	**0.76 (0.64,0.92)**
T1	<32.8	Ref	Ref
T2	32.9–49.4	**0.76 (0.57,0.99)**	**0.71 (0.53,0.96)**
T3	>49.5	0.80 (0.61,1.1)	**0.67 (0.49,0.92)**
Quinolinic acid (QA)			
Continuous^b^	458.0 (361.0–603.0)	**1.88 (1.61,2.18)**	**1.31 (1.05,1.63)**
T1	<390.0	Ref	Ref
T2	391.0–544.0	1.37 (0.99,1.91)	1.10 (0.77,1.56)
T3	>545.0	**2.43 (1.81,3.26)**	1.40 (0.98,2.00)

*Note*: Trp, Kyn, PA, Kyn/Trp ratio: Total events: 290; total patients: 2088. QA, XA, KA: Total events: 290; total patients: 2089. AA, HK, HAA: Total events: 288; total patients: 2073. *p* values <0.05 are statistically significant and are indicated in bold text.

Abbreviations: Adj., adjusted; CI, confidence interval; HR, hazard ratio; IQR, interquartile range; T, tertile.

^a^ Model adjusted using Cox proportional hazards regression for age, sex, tumor stage, tumor site, creatinine, and study cohort. Tertiles were derived for the overall study population.

^b^ Analysis performed using log2‐transformed concentrations. Thus, hazard ratios represent a doubling in concentrations.

Stratified analyses by tumor site did not show any substantial differences between descending colon, ascending colon, and rectal cancer (Table S4). We only observed significant heterogeneity in the associations of QA with death: a doubling of QA concentration was associated with a 1.62‐fold increase in risk of death in patients diagnosed with ascending colon cancer (HR_ascending colon_ = 1.62; 95%CI:1.16,2.28) while no statistically significant association was observed with risk of death in patients diagnosed with descending colon and rectal cancer (HR_descending colon_ = 1.02; 95%CI:0.62,1.68; HR_rectal_ = 1.13; 95%CI:0.80,1.60; *P*_interaction_ = 0.04). Stratified analyses by tumor stage revealed similar trends across tumor stages, however, some significant differences were found (Table S5). For example, while kynurenine was not significantly associated with all‐cause mortality when stage I–III CRC patients were combined, in stratified analyses we found that kynurenine was associated with a 3‐fold increased risk of death among patients with stage I CRC and there was a lower, statistically not significant association found for patients with stage II CRC (HR_stageI_ = 3.13; 95%CI:1.15,8.54; HR_stageII_ = 1.66; 95%CI:0.81,3.41; HR_stageIII_ = 0.81; 95%CI:0.46,1.45 *p*_interaction_ <0.0001). On the other hand, a doubling in PA concentration was associated with a reduced risk of death among stage II and III CRC patients (HR_stageI_ = 1.13; 95%CI:0.71,1.80; HR_stageII_ = 0.68; 95%CI:0.48,0.95; HR_stageIII_ = 0.72; 95%CI:0.56,0.92; *p*_interaction_ = 0.02), and a doubling in HAA concentration was associated with a reduced risk of death among stage III CRC patients (HR_stageI_ = 1.04; 95%CI:0.59,1.83; HR_stageII_ = 0.94; 95%CI:0.63,1.41; HR_stageIII_ = 0.68; 95%CI:0.50,0.93; *p*_interaction_ = 0.003). A doubling in the Kyn/Trp ratio was associated with increased risk of death among stage I and stage II CRC patients and a similar, but less strong and not statistically significant, trend was seen for patients with stage III CRC (HR_stageI_ = 6.78; 95%CI:2.88,16.0; HR_stageII_ = 2.51; 95%CI:1.44,4.38; HR_stageIII_ = 1.39; 95%CI:0.89,2.17; *p*_interaction_ = 0.003).

Several studies reported that kynurenine and the Kyn/Trp ratio is increased in individuals with obesity^34^, ^35^ and in this study, we found a similar trend for CRC patients (Table S2). To explore whether there are any interactions between obesity and tryptophan–kynurenine metabolites on CRC all‐cause mortality, stratified analysis by BMI was performed. No statistically significant differences between normal weight, overweight, and obese CRC patients were observed (Table S6). However, interestingly, kynurenine was associated with a 2.64‐fold increased risk of death among obese CRC patients, while no significant association was observed for normal weight and overweight CRC patients (HR_obese_ = 2.64; 95%CI:1.09,6.39; HR_overweight_ = 1.00; 95%CI:0.54,1.85; HR_normal weight_ = 0.52; 95%CI:0.32,0.87; *p*_interaction_ = 0.11).

When stratified by study cohort, limited heterogeneity was found in the associations between tryptophan and kynurenine in relation to all‐cause mortality (Table S7). Higher concentrations of tryptophan were associated with a reduced risk of death in the COLON study, while associations in other cohorts were quite heterogenous. Higher kynurenine concentrations tended to be associated with a reduced risk of death in the COLON study, while in other cohorts, higher kynurenine concentrations seemed to be associated with a higher risk of death. No large differences between cohorts were found in the associations for other tryptophan–kynurenine metabolites and all‐cause mortality. The stratified analyses for the ColoCare HCI cohort need to be interpreted with caution as the number of patients is relatively low. In general, the associations observed for the total study population were most in line with the associations within the COLON cohort, which represents around half of the total study population.

In sensitivity analyses, excluding patients who died within 60 days after blood draw (*n* = 27 patients), similar associations were observed (Table S8). Additional adjustment for BMI, smoking history or physical activity level showed similar associations for all‐cause mortality (Table S9). No substantial differences in our findings were observed after additional adjustment for neoadjuvant or adjuvant therapy (Table S10).

## 4 DISCUSSION

To our knowledge, this is the first study investigating associations of circulating tryptophan–kynurenine pathway metabolites with all‐cause mortality in stage I–III CRC patients.

Our results showed that a higher plasma Kyn/Trp ratio was associated with an increased risk of death in CRC patients. It has to be noted that the Kyn/Trp ratio is not fully reflecting IDO1 activity in vivo,^10^ but there is evidence for the prognostic value of the Kyn/Trp ratio in glioblastoma, nasopharyngeal carcinoma and non‐small cell lung cancer patients.^36^, ^37^, ^38^ IDO1 is upregulated by proinflammatory cytokines such as interleukin‐6 and IFN‐γ, while it is inhibited by anti‐inflammatory cytokines such as interleukin‐4, suggesting that IDO1 activity is determined by the balance between pro‐ and anti‐inflammatory cytokines.^39^ Therefore, our results support the notion that systemic inflammation may predispose to increased risk of all‐cause mortality in CRC patients, as has been observed for other biomarkers of inflammation, specifically interleukin‐8 and high‐sensitivity C‐reactive protein.^40^

As multimorbidity is common in CRC patients,^41^ associations between tryptophan metabolites and all‐cause mortality may be related to other health conditions apart from CRC recurrence. Alterations in tryptophan metabolism are linked to obesity, CVD, and type 2 diabetes.^3^, ^4^, ^5^, ^6^ In line with our findings, in a population‐based cohort study of ~7000 adults, a higher plasma Kyn/Trp ratio was associated with a higher risk of all‐cause and cause‐specific mortality due to cancer or CVD while higher plasma tryptophan levels were associated with a decreased risk of all‐cause and cause‐specific mortality.^42^ Specifically, for CVD, it has been shown that a higher Kyn/Trp ratio was associated with an increased risk of coronary events in older adults without previous coronary heart disease.^3^ Potentially, a higher Kyn/Trp ratio, induced by IFN‐γ, is an indicator of inflammatory processes related to atherosclerosis,^43^ or related to impairments in insulin sensitivity.^44^ Therefore, both cancer‐related and other mechanistic routes (via cardiometabolic health) may explain our observations that link tryptophan–kynurenine pathway metabolites to all‐cause mortality in CRC patients.

Only limited evidence is available that may explain the underlying mechanisms observed in our cohort. The metabolites XA and HK are shown to modulate inflammation, but their pro‐ or anti‐inflammatory and pro‐ or anti‐oxidative function is highly depending on the experimental setting.^11^ QA is mainly studied in context of the central nervous system, is thought to have neurotoxic effects and plays a key role in promotion of neuroinflammation.^45^ Nevertheless, potential mechanistic routes underlying the observed associations between tryptophan–kynurenine metabolites and all‐cause mortality remain speculative.

We observed some heterogeneity in the associations between tryptophan and kynurenine in relation to all‐cause mortality when stratified for cohort. However, the sample size of the separate cohorts is too limited to draw robust conclusions. We further stratified our analyses by tumor site since there is accumulating evidence on heterogeneity between colon and rectal cancer with regards to prognosis,^46^ indicating that colon and rectal cancer have distinct etiologies as well as prognostic markers. Furthermore, plasma tryptophan metabolic profiles were shown to significantly differ between patients with colon and rectal cancer.^30^ We only observed significant heterogeneity for associations between concentrations of QA and all‐cause mortality, in particular, higher concentrations of QA were associated with a significant increased risk of death among patients with tumors located in the ascending colon. We observed linear trends for associations across tumor stages. While associations for HAA, XA, PA were most pronounced in stages II and III of CRC, associations for kynurenine, QA and the Kyn/Trp ratio were most pronounced in CRC patients with stage I, suggesting that these metabolites and the Kyn/Trp ratio may serve as prognostic indicators in early stages of CRC. These results also indicate that metabolites located along the tryptophan–QA–nicotinamide adenine dinucleotide (NAD^+^) axis may be elevated, leading to increased production of NAD^+^, which is required for cancer cells to meet their higher demand for adenosine triphosphate (ATP) and promote tumor growth.^47^ However, kynurenine pathway‐driven NAD^+^ production in cancer progression still remains poorly understood. Further research is therefore needed (1) to confirm these stage‐specific differences and (2) to elucidate the biological mechanisms as to why associations between kynurenine metabolites and all‐cause mortality may differ across subgroups of CRC patients, for example, with different tumor stage.

We acknowledge both the strengths and limitations of our study. We utilized data from various European and US cohort studies comprising a large number of geographically diverse CRC patients. However, since these cohort studies do not uniformly collect information regarding CRC‐specific or other causes of death due to privacy regulations, we were unable to explore the potential association between tryptophan–kynurenine pathway metabolite concentrations and CRC‐specific death or account for competing risks of death. Since technical factors (e.g., serum/plasma blood samples and fasted/nonfasted) were study cohort specific, we cannot fully exclude the possibility of residual confounding by these factors. However, we adjusted for study cohort and our results persisted, even after adjustment for other covariates, and also remained consistent in sensitivity analyses. All cohorts involved in this study have implemented standardized operating procedures for active follow‐up of patients, which involves capturing clinical outcomes such as all‐cause mortality through medical chart abstraction, death registries, or external general practitioners. We addressed the most relevant confounders, nevertheless, like in other observational studies, the possibility of residual confounding cannot be fully excluded, for example potential reverse causation due to underlying comorbidities affecting tryptophan–kynurenine metabolites.

In conclusion, this study provides first evidence that circulating tryptophan–kynurenine pathway metabolites are associated with all‐cause mortality in patients with stage I–III CRC. By identifying circulating tryptophan–kynurenine pathway metabolites in relation to mortality, our study provides leads for further research focusing on prognostic relevance of these metabolites in non‐metastatic CRC patients. This can help in facilitating early detection of high‐risk patients and personalizing treatment and surveillance plans. Furthermore, our findings contribute to the understanding of the underlying mechanisms in CRC progression and prognosis, which provides new therapeutic targets within the tryptophan–kynurenine pathway. Future studies will be needed to assess whether dietary or pharmacological interventions may improve CRC prognosis through modulation of the tryptophan–kynurenine pathway.

## AUTHOR CONTRIBUTIONS

**Victoria Damerell:** Conceptualization; data curation; formal analysis; methodology; software; writing – original draft. **Niels Klaassen‐Dekker:** Conceptualization; data curation; formal analysis; methodology; software; writing – original draft. **Stefanie Brezina:** Data curation. **Jennifer Ose:** Data curation; formal analysis. **Arve Ulvik:** Data curation; software. **Eline H. van Roekel:** Data curation. **Andreana N. Holowatyj:** Data curation; formal analysis. **Andreas Baierl:** Data curation. **Jürgen Böhm:** Investigation. **Martijn J. L. Bours:** Conceptualization. **Hermann Brenner:** Investigation. **Johannes H. W. de Wilt:** Data curation. **William M. Grady:** Investigation. **Michael Hoffmeister:** Investigation. **Tengda Lin:** Formal analysis. **Peter Schirmacher:** Investigation. **Alexis B. Ulrich:** Investigation. **Christopher I. Li:** Investigation. **Andrea Gsur:** Conceptualization; funding acquisition. **Ellen Kampman:** Conceptualization; funding acquisition. **Martin Schneider:** Conceptualization. **Per M. Ueland:** Conceptualization; data curation; funding acquisition; software. **Matty P. Weijenberg:** Conceptualization; funding acquisition. **Cornelia M. Ulrich:** Conceptualization; funding acquisition; investigation; project administration; resources; supervision. **Dieuwertje E. Kok:** Conceptualization; investigation; project administration; resources; supervision. **Biljana Gigic:** Conceptualization; formal analysis; investigation; project administration; resources; software; supervision.

## FUNDING INFORMATION

The FOCUS Consortium is an ERA‐NET (European Research Area Network) on Translational Cancer Research (TRANSCAN) project funded by the local research funding agencies (German Ministry of Education and Research, Germany, 01KT1503; Dutch Cancer Society with grant number UW2014‐6877, the Netherlands; FWF Austrian Science Fund, Austria (API02104FW); Research Council Norway/Norwegian Cancer Society (RNC), Norway). The COLON study is further sponsored by Wereld Kanker Onderzoek Fonds, including funds from grant 2014/1179 as part of the World Cancer Research Fund International Regular Grant Programme; Alpe d'Huzes/Dutch Cancer Society (UM 2012–5653, UW 2013–5927, UW 2015–7946); and ERA‐NET on Translational Cancer Research (TRANSCAN/Dutch Cancer Society: UW2013‐6397 and the Netherlands Organization for Health Research and Development [ZonMw], the Netherlands). The EnCoRe study was supported by grants from the Stichting Alpe d'HuZes within the research program ‘Leven met kanker’ of the Dutch Cancer Society (Grant No. UM‐2010‐4867 and UM‐2012‐5653), and by grants from Kankeronderzoekfonds Limburg as part of Health Foundation Limburg (Grant No. 00005739), and by a grant from Wereld Kanker Onderzoek Fonds (WKOF), as part of the World Cancer Research Fund International grant programme (grant number 2016/1620). VD was funded by the German Ministry of Education and Research project PerMiCCion (01KD2101D), the Rahel‐Goitein‐Straus‐Program, Medical Faculty, Heidelberg University, and the Heidelberger Stiftung Chirurgie, Heidelberg University Hospital. BG was funded by the ERA‐NET on Translational Cancer Research (TRANSCAN), the German Ministry of Education and Research projects 01KT1503 and 01KD2101D, the National Institutes of Health/National Cancer Institute (NIH/NCI) projects R01 CA189184 and U01 CA206110, the Stiftung LebensBlicke, and the Matthias‐Lackas Foundations. EvR was funded by the Wereld Kanker Onderzoek Fonds (WKOF), part of the World Cancer Research Fund International grant programme (grant number 2016/1620). MB was funded by Alpe d'HuZes within the research program ‘Leven met kanker’ of the Dutch Cancer Society (Grant No. UM‐2012‐5653). NKD was financially supported by VLAG Graduate School. ANH was supported by the National Institutes of Health under Ruth L. Kirschstein National Research Service Award T32 HG008962 from the National Human Genome Research Institute. NH was funded by the TRANSCAN project 01KT1512. CMU and JO were funded by the Huntsman Cancer Foundation, and NIH/NCI projects R01 CA189184, R01 CA207371, U01 CA206110, U01 CA206110 and P30 CA042014. National Insitutes of Health grants (P30 CA15704, U01 CA152756, R01 CA194663, R01 CA220004, P01 CA077852), Rodger C. Haggitt Endowed Chair, R.A.C.E. Charities, Cottrell Family Fund, Listwin Family Foundation, Seattle Translational Tumor Research program. WMG was funded by Fred Hutchinson Cancer Research Center. SB was funded by the ERA‐NET on Translational Cancer Research (TRANSCAN) project FOCUS I2104‐B26 and the ERA‐NET on Translational Cancer Research (TRANSCAN) project MetaboCCC I 1578–B19.

## CONFLICT OF INTEREST STATEMENT

As Executive Director of the Comprehensive Cancer Center at Huntsman Cancer Institute (Salt Lake City, Utah), Cornelia M. Ulrich formally oversees research funded by several pharmaceutical companies. However, she does not direct those research efforts and has not received funding directly herself that would constitute a conflict to the current study. Dr. Holowatyj reports receiving grants from the National Institutes of Health (NIH), Dalton Family Foundation, Pfizer, the American Cancer Society and the Appendix Cancer Pseudomyxoma Peritonei (ACPMP) Research Foundation outside the submitted work; chairing the scientific advisory board for the ACPMP Research Foundation; and personal fees from MJH Life Sciences and Bayer AG outside this study. Dr. Grady: consultanting for Guardant Health, Freenome, Karius and Diacarta; research support from LuidDx. The other authors have no conflicts of interest to declare.

## ETHICS STATEMENT

Each study site protocol was approved by the respective institutional review board and all patients provided an informed consent.

## DISCLAIMER

Where authors are identified as personnel of the International Agency for Research on Cancer/World Health Organization, the authors alone are responsible for the views expressed in this article and they do not necessarily represent the decisions, policy or views of the International Agency for Research on Cancer/World Health Organization.

## Supporting information


**DATA S1.** Supplementary information.

## Data Availability

The data that support the findings of this study are available from the corresponding author upon reasonable request. For further questions please contact colocarestudy_admin@hci.utah.edu.
