# Supplementary material for: Circulating tryptophan–kynurenine pathway metabolites are associated with all‐cause mortality among patients with stage I–III colorectal cancer
Source: Int J Cancer. 2024 Sep 23;156(3):552–65. doi: 10.1002/ijc.35183 (PMC11621991; doi:10.1002/ijc.35183)
Supplement: Supplementary file 1 — DATA S1. Supplementary information. [file IJC-156-552-s001.docx]

**Circulating tryptophan-kynurenine pathway metabolites are associated with all-cause mortality among patients with stage I–III colorectal cancer**

Victoria Damerell, Niels Klaassen-Dekker, Stefanie Brezina, Jennifer Ose, Arve Ulvik, Eline H van Roekel, Andreana N Holowatyj, Andreas Baierl, Jürgen Böhm, Martijn JL Bours, Hermann Brenner, Johannes HW de Wilt, William M Grady, Nina Habermann, Michael Hoffmeister, Pekka Keski-Rahkonen, Janna L Koole, Tengda Lin, Peter Schirmacher, Petra Schrotz-King, Alexis B Ulrich, Fränzel JB van Duijnhoven, Christy A Warby, David Shibata, Adetunji T Toriola, Jane C Figueiredo, Erin M Siegel, Christopher I Li, Andrea Gsur, Ellen Kampman, Martin Schneider, Per M Ueland, Matty P Weijenberg, Cornelia M Ulrich, Dieuwertje E Kok, and Biljana Gigic, FOCUS Consortium.

**Table of contents**

**Supplementary Table S1 page 2**

**Supplementary Table S2 page 4**

**Supplementary Table S3 page 8**

**Supplementary Table S4 page 11**

**Supplementary Table S5 page 13**

**Supplementary Table S6 page 15**

**Supplementary Table S7 page 17**

**Supplementary Table S8 page 20**

**Supplementary Table S9 page 22**

**Supplementary Table S10 page 24**

| Table S1. Demographic and primary tumor characteristics of the study population stratified by cohort: FOCUS Consortium.^1^ | | | | | | | |
| --- | --- | --- | --- | --- | --- | --- | --- |
|  |  | **Cohorts** | | | | | |
|  | **Study population** | **COLON** | **EnCoRe** | **ColoCare FHCRC** | **ColoCare HCI** | **ColoCare HD** | **CORSA** |
| Number of participants, n (%) | **2102** | **1118** | **297** | **131** | **68** | **271** | **217** |
| Sex  Female, n (%) | 754 (35.9) | 404 (36.1) | 97 (32.7) | 61 (46.6) | 27 (39.7) | 90 (33.2) | 75 (34.6) |
| Male, n (%) | 1348 (64.1) | 714 (63.9) | 200 (67.3) | 70 (53.4) | 41 (60.3) | 181 (66.8) | 142 (65.4) |
| Age at diagnosis (years), median | 65.4 (10.2) | 66.2 | 66.6 | 58.0 | 61.4 | 64.2 | 67.3 |
| Body mass index (kg/m^2^), median | 27.1 (4.6) | 26.5 | 28.2 | 28.9 | 29.2 | 26.7 | 27.7 |
| Smoking history, n (%) |  |  |  |  |  |  |  |
| Current | 258 (12.3) | 123 (11.4) | 39 (13.1) | 8 (6.1) | 4 (5.9) | 47 (17.3) | 37 (17.1) |
| Former | 1060 (50.4) | 642 (57.4) | 160 (53.9) | 44 (33.6) | 18 (26.5) | 120 (44.3) | 76 (35.0) |
| Never | 672 (31.9) | 317 (28.3) | 92 (31.0) | 48 (36.6) | 36 (52.9) | 82 (30.3) | 97 (44.6) |
| Unknown/missing | 112 (5.3) | 36 (3.2) | 6 (2.0) | 31 (23.7) | 10 (14.7) | 22 (8.1) | 7 (3.2) |
| Adherence to physical activity guidelines*, n (%) |  |  |  |  |  |  |  |
| Yes | 1135 (54.0) | 791 (70.8) | 217 (73.1) | 32 (24.4) | 17 (25.0) | 78 (28.8) | 0 (0.0) |
| No | 617 (29.4) | 288 (25.7) | 74 (24.9) | 53 (40.5) | 40 (58.8) | 162 (59.8) | 0 (0.0) |
| Unknown | 350 (16.6) | 39 (3.5) | 6 (2.0) | 46 (35.1) | 11 (16.2) | 31 (11.4) | 217 (100.0) |
| Tumor stage**, n (%) |  |  |  |  |  |  |  |
| I | 558 (26.6) | 280 (25.0) | 86 (29.0) | 30 (22.9) | 16 (23.5) | 65 (24.0) | 81 (37.3) |
| II | 605 (28.8) | 327 (29.2) | 59 (19.9) | 42 (32.1) | 18 (26.5) | 107 (39.5) | 52 (24.0) |
| III | 848 (40.3) | 462 (41.3) | 142 (47.8) | 59 (45.0) | 34 (50.0) | 99 (36.5) | 52 (24.0) |
| Unknown/unspecified | 91 (4.3) | 49 (4.5) | 10 (3.3) | 0 (0.0) | 0 (0.0) | 0 (0.0) | 32 (14.7) |
| Tumor site, n (%) |  |  |  |  |  |  |  |
| Ascending colon | 598 (28.4) | 329 (29.4) | 75 (25.3) | 29 (22.1) | 24 (35.3) | 72 (26.6) | 69 (31.8) |
| Descending colon | 663 (31.5) | 388 (34.7) | 104 (35.0) | 34 (26.0) | 16 (23.5) | 59 (21.8) | 62 (28.6) |
| Rectosigmoid junction/rectum | 803 (38.2) | 373 (33.3) | 118 (39.7) | 67 (51.1) | 23 (33.8) | 140 (51.7) | 82 (37.8) |
| Unknown | 38 (1.8) | 28 (2.6) | 0 (0.0) | 1 (0.8) | 5 (7.4) | 0 (0.0) | 4 (1.8) |
| Neoadjuvant treatment, n (%) |  |  |  |  |  |  |  |
| No | 1586 (75.5) | 837 (74.9) | 216 (72.7) | 88 (67.2) | 46 (67.6) | 206 (76.0) | 193 (88.9) |
| Yes | 470 (24.5) | 252 (22.5) | 81 (27.9) | 42 (32.1) | 15 (22.1) | 63 (23.2) | 17 (7.8) |
| Unknown | 46 (2.1) | 29 (2.6) | 0 (0.0) | 1 (0.7) | 7 (10.3) | 2 (0.8) | 7 (3.3) |
| Type of neoadjuvant treatment, n (%) |  |  |  |  |  |  |  |
| Chemotherapy | 13 (2.8) | 5 (2.0) | 0 (0.0) | 0 (0.0) | 3 (20.0) | 2 (3.2) | 3 (17.6) |
| Radiation therapy | 180 (38.3) | 137 (54.5) | 20 (24.6) | 1 (2.4) | 1 (6.7) | 18 (28.6) | 3 (17.6) |
| Chemoradiation | 277 (58.9) | 110 (43.5) | 61 (75.4) | 41 (97.6) | 11 (73.3) | 43 (68.3) | 11 (64.8) |
| Unknown | 0 (0.0) |  |  |  |  |  |  |
| Adjuvant treatment, n (%) |  |  |  |  |  |  |  |
| No | 1204 (57.3) | 783 (70.0) | 199 (67.0) | 71 (54.2) | 16 (23.5) | 0 (0) | 149 (68.7) |
| Yes | 765 (36.3) | 253 (22.5) | 97 (32.6) | 57 (43.5) | 29 (42.6) | 256 (94.5) | 59 (27.1) |
| Unknown | 133 (6.3) | 82 (7.5) | 1 (0.4) | 3 (2.3) | 23 (33.9) | 15 (5.5) | 9 (4.2) |
| Type of adjuvant treatment, n (%) |  |  |  |  |  |  |  |
| Chemotherapy | 556 (26.5) | 236 (21.1) | 95 (32.0) | 65 (49.6) | 29 (42.6) | 85 (31.4) | 46 (21.2) |
| Radiation therapy | 170 (8.1) | 2 (0.2) | 1 (0.3) | 0 (0.0) | 0 (0.0) | 164 (60.5) | 3 (1.4) |
| Chemoradiation | 31 (1.5) | 7 (0.6) | 1 (0.3) | 6 (4.6) | 0 (0.0) | 7 (2.6) | 10 (4.6) |
| Unknown | 8 (0.4) | 8 (0.7) | 0 (0.0) | 0 (0.0) | 0 (0.0) | 0 (0.0) | 0 (0.0) |
| Vital status |  |  |  |  |  |  |  |
| Alive | 1812 (86.2) | 977 (87.4) | 269 (90.6) | 107 (81.7) | 63 (92.6) | 242 (89.3) | 154 (71.0) |
| Deceased | 290 (13.8) | 141 (12.6) | 28 (9.4) | 24 (18.3) | 5 (7.4) | 29 (10.7) | 63 (29.0) |
| ^1^Values are presented as n (%) or mean and standard deviation (SD). *Self-reported engagement in at least 150 min per week of moderate-to-vigorous physical activity. ** Sixteen patients had a CRC that could not be distinguished between stage I–II or stage II–III disease. Abbreviations: CRC, Colorectal cancer; COLON, Colorectal cancer: Longitudinal, Observational study on Nutritional and lifestyle factors that may influence colorectal tumor recurrence, survival and quality of life Study; CORSA, Colorectal Cancer Study of Austria; EnCoRe, Energy for life after ColoRectal cancer; FHCRC, Fred Hutchinson Cancer Research Center; HCI, Huntsman Cancer Institute; HD, Heidelberg. | | | | | | | |

| Table S2. Baseline characteristics stratified by tertiles of tryptophan, kynurenine and the Kyn/Trp ratio. | | | | | | |
| --- | --- | --- | --- | --- | --- | --- |
|  |  |  |  | **Tryptophan** |  |  |
|  | **Median concentration (IQR)^1^** | **Total** | **T1** | **T2** | **T3** | **p-value^2^** |
| Age (years)  <50 n (%)  50-69 n (%)  >70 n (%) | 61.2 (54.9,70.2)  62.5 (53.3,71.4)  58.7 (49.9,69.7) | 153  1226  721 | 43 (28.1)  367 (29.9)  290 (40.2) | 61 (39.9)  422 (34.4)  217 (30.1) | 49 (32.0)  437 (35.7)  214 (29.7) | <0.0001 |
| Sex n (%)  Female  Male | 57.2 (49.4,66.0)  63.4 (54.7,72.4) | 754  1346 | 331 (43.9)  369 (27.4) | 254 (33.7)  446 (33.1) | 169 (22.4)  531 (39.5) | <0.0001 |
| Tumor stage n (%)  I  II  III | 62.7 (53.3,71.9)  59.0 (50.2,69.7)  61.7 (53.2,70.2) | 556  605  848 | 173 (31.1)  231 (38.2)  267 (31.5) | 180 (32.4)  196 (32.4)  297 (35.0) | 203 (36.5)  178 (29.4)  284 (33.5) | 0.02 |
| Tumor site n (%)  ascending colon  descending colon  rectum | 58.5 (50.1,68.4)  62.2 (53.7,71.2)  62.1 (53.8,71.2) | 598  662  802 | 244 (40.8)  198 (29.9)  241 (30.0) | 192 (32.1)  227 (34.3)  277 (34.6) | 162 (27.1)  237 (35.8)  284 (35.4) | <0.0001 |
| BMI  underweight n (%)  normal weight n (%)  overweight n (%)  obese n (%) | 57.5 (42.6,71.4)  59.6 (50.3,68.8)  61.7 (53.6,70.6)  62.5 (53.2,72.0) | 19  709  895  443 | 8 (42.1)  268 (37.8)  272 (30.4)  138 (31.2) | 6 (31.6)  218 (30.7)  326 (36.4)  141 (31.8) | 5 (26.3)  223 (31.5)  297 (33.2)  164 (37.0) | 0.02 |
| Smoking history n (%)  Current  Former  Never | 60.1 (50.9,69.9)  61.9 (53.0,71.5)  59.7 (52.0,69.4) | 258  1059  671 | 86 (33.3)  340 (32.1)  234 (34.9) | 91 (35.3)  347 (32.8)  233 (34.7) | 81 (31.4)  372 (35.1)  204 (30.4) | 0.33 |
| Physical activity* n (%)  Yes  No | 62.3 (53.6,71.6)  59.3 (50.2,69.4) | 1134  616 | 338 (29.8)  238 (38.6) | 386 (34.0)  196 (31.8) | 410 (36.2)  182 (29.6) | 0.0005 |
|  |  |  |  | **Kynurenine** |  |  |
|  | **Median concentration (IQR)^1^** | **Total** | **T1** | **T2** | **T3** | **p-value^2^** |
| Age (years)  <50 n (%)  50-69 n (%)  >70 n (%) | 1.5 (1.3,1.7)  1.6 (1.4,1.8)  1.8 (1.5,2.1) | 153  1226  721 | 80 (52.3)  464 (37.9)  156 (21.6) | 56 (36.6)  423 (34.5)  221 (30.7) | 17 (11.1)  339 (27.7)  344 (47.7) | <0.0001 |
| Sex n (%)  Female  Male | 1.6 (1.3,1.8)  1.7 (1.5,1.9) | 754  1346 | 322 (42.7)  378 (28.1) | 232 (30.8)  468 (34.8) | 200 (26.5)  500 (37.2) | <0.0001 |
| Tumor stage n (%)  I  II  III | 1.6 (1.4,1.9)  1.7 (1.4,1.9)  1.7 (1.4,1.9) | 556  605  848 | 184 (33.1)  192 (31.7)  294 (34.7) | 182 (32.7)  193 (36.4)  298 (35.1) | 190 (34.2)  220 (36.4)  256 (30.2) | 0.17 |
| Tumor site n (%)  ascending colon  descending colon  rectum | 1.7 (1.4,1.9)  1.7 (1.4,1.9)  1.6 (1.4,1.9) | 598  662  802 | 186 (31.1)  211 (31.9)  288 (35.9) | 191 (31.9)  230 (34.7)  270 (33.7) | 221 (37.0)  221 (33.4)  244 (30.4) | 0.09 |
| BMI  underweight n (%)  normal weight n (%)  overweight n (%)  obese n (%) | 1.4 (1.3,1.7)  1.5 (1.3,1.8)  1.7 (1.5,1.9)  1.8 (1.5,2.0) | 19  709  895  443 | 10 (52.6)  312 (44.0)  266 (29.7)  97 (21.9) | 5 (26.3)  209 (29.5)  338 (37.8)  141 (31.8) | 4 (21.1)  188 (26.5)  291 (32.5)  205 (46.3) | <0.0001 |
| Smoking history n (%)  Current  Former  Never | 1.6 (1.31,1.73)  1.7 (1.44,1.93)  1.63 (1.41,1.89) | 258  1059  671 | 122 (47.3)  322 (30.4)  224 (33.4) | 84 (32.6)  354 (33.4)  230 (34.3) | 52 (20.1)  383 (36.2)  217 (32.3) | <0.0001 |
| Physical activity* n (%)  Yes  No | 1.63 (1.41,1.87)  1.66 (1.44,1.93) | 1134  616 | 394 (34.7)  190 (30.8) | 389 (34.3)  211 (34.3) | 351 (31.0)  215 (34.9) | 0.15 |
|  |  |  |  | **Kyn/Trp ratio** |  |  |
|  | **Median concentration (IQR)^1^** | **Total** | **T1** | **T2** | **T3** | **p-value^2^** |
| Age (years)  <50 n (%)  50-69 n (%)  >70 n (%) | 0.024 (0.021,0.026)  0.025 (0.022,0.030)  0.030 (0.025,0.037) | 153  1226  721 | 79 (51.6)  479 (39.1)  142 (19.7) | 54 (35.3)  435 (35.5)  211 (29.3) | 20 (13.1)  312 (25.5)  368 (51.0) | <0.0001 |
| Sex n (%)  Female  Male | 0.027 (0.022,0.033)  0.026 (0.023,0.032) | 754  1346 | 240 (31.8)  460 (34.2) | 248 (32.9)  452 (33.6) | 266 (35.3)  434 (32.2) | 0.33 |
| Tumor stage n (%)  I  II  III | 0.026 (0.023,0.031)  0.027 (0.023,0.034)  0.026 (0.023,0.031) | 556  605  848 | 187 (33.6)  193 (31.9)  291 (34.3) | 203 (36.5)  175 (28.9)  290 (34.2) | 166 (29.9)  237 (39.2)  267 (31.5) | 0.005 |
| Tumor site n (%)  ascending colon  descending colon  rectum | 0.028 (0.024,0.034)  0.026 (0.022,0.031)  0.025 (0.022,0.031) | 598  662  802 | 161 (26.9)  231 (34.9)  297 (37.0) | 181 (30.3)  227 (34.3)  280 (34.9) | 256 (42.8)  204 (30.8)  225 (29.1) | <0.0001 |
| BMI  underweight n (%)  normal weight n (%)  overweight n (%)  obese n (%) | 0.026 (0.022,0.029)  0.025 (0.022,0.031)  0.026 (0.023,0.032)  0.028 (0.024,0.034) | 19  709  895  443 | 9 (47.4)  286 (40.3)  286 (32.0)  108 (24.4) | 6 (31.6)  213 (30.0)  326 (36.4)  145 (32.7) | 4 (21.1)  210 (29.6)  283 (31.6)  190 (42.9) | <0.0001 |
| Smoking history n (%)  Current  Former  Never | 0.025 (0.021,0.030)  0.026 (0.023,0.032)  0.027 (0.023,0.032) | 258  1059  671 | 111 (43.0)  340 (32.1)  215 (32.0) | 82 (31.8)  362 (34.2)  221 (32.9) | 65 (25.2)  357 (33.7)  235 (35.0) | 0.007 |
| Physical activity* n (%)  Yes  No | 0.026 (0.022,0.031)  0.027 (0.023,0.034) | 1134  616 | 406 (35.8)  176 (28.6) | 401 (35.4)  205 (33.3) | 327 (28.8)  235 (38.2) | 0.0002 |
| ^1^Concentration in nanomolar/liter. ^2^Chi-square tests were used to compare differences between groups. *Self-reported engagement in at least 150 min per week of moderate-to-vigorous physical activity. Abbreviations: BMI, body mass index; IQR, interquartile range; Kyn/Trp ratio, kynurenine-to-tryptophan ratio; T, tertile. | | | | | | |

| Table S3. Associations between tryptophan-kynurenine pathway metabolites and all-cause mortality among patients with stage I-III CRC (n=2,102). Tertiles derived by study cohort. | | | |
| --- | --- | --- | --- |
|  |  | **All-cause mortality** | |
| Biomarker | **Median (IQR) concentration** | **Crude HR**  **(95% CI)** | **Adj. HR^1^**  **(95% CI)** |
| Tryptophan |  |  |  |
| Continuous^a^ | 61.2 (52.3-70.7) | 0.43 (0.32,0.58) | **0.56 (0.41,0.77)** |
| T1 | <56.3 | ref | ref |
| T2 | 51.5-64.2 | 0.68 (0.52,0.88) | **0.75 (0.56,0.99)** |
| T3 | >65.1 | 0.56 (0.41,0.74) | **0.63 (0.46,0.86)** |
| Kynurenine |  |  |  |
| Continuous^a^ | 1.6 (1.4-1.9) | 2.37 (1.73,3.26) | 1.28 (0.86,1.92) |
| T1 | <1.6 | ref | ref |
| T2 | 1.4-1.9 | 1.05 (0.77,1.43) | 0.88 (0.64,1.22) |
| T3 | >1.8 | 1.77 (1.33,2.34) | 1.07 (0.77,1.49) |
| Kyn/Trp ratio |  |  |  |
| Continuous^a^ | 0.027 (0.022-0.032) | 3.09 (2.44,3.90) | **2.07 (1.52,2.83)** |
| T1 | <0.025 | ref | ref |
| T2 | 0.024-0.032 | 1.31 (0.94,1.82) | 1.11 (0.79,1.60) |
| T3 | >0.029 | 2.55 (1.89,3.44) | **1.63 (1.15,2.31)** |
| Kynurenic acid (KA) |  |  |  |
| Continuous^a^ | 47.7 (36.9-62.1) | 1.32 (1.10,1.58) | 0.87 (0.68,1.12) |
| T1 | <43.7 | ref | ref |
| T2 | 33.8-60.8 | 0.83 (0.61,1.11) | 0.70 (0.51,0.97) |
| T3 | >53.4 | 1.29 (0.99,1.69) | 0.75 (0.53,1.06) |
| Anthranilic acid (AA) |  |  |  |
| Continuous^a^ | 16.2 (12.8-21.6) | 1.38 (1.15,1.65) | 0.82 (0.72,1.17) |
| T1 | <21.8 | ref | ref |
| T2 | 12.5-30.0 | 1.13 (0.84,1.52) | 0.82 (0.59,1.13) |
| T3 | >17.2 | 1.52 (1.14,2.01) | 0.92 (0.67,1.27) |
| 3-Hydroxykynurenine (HK) |  |  |  |
| Continuous^a^ | 45.0 (35.0-58.4) | 2.10 (1.80,2.46) | **1.80 (1.47,2.21)** |
| T1 | <42.8 | ref | ref |
| T2 | 33.2-60.4 | 1.13 (0.81,1.56) | 0.97 (0.69,1.38) |
| T3 | >49.4 | 2.27 (1.70,3.03) | **1.53 (1.08,2.15)** |
| Xanthurenic acid (XA) |  |  |  |
| Continuous^a^ | 13.4 (8.8-18.8) | 0.82 (0.72,0.92) | **0.74 (0.64,0.85)** |
| T1 | <11.5 | ref | ref |
| T2 | 7.6-18.0 | 0.76 (0.58,0.99) | **0.75 (0.56,0.99)** |
| T3 | >13.0 | 0.70 (0.53,0.93) | **0.59 (0.43,0.82)** |
| 3-Hydroxyanthranilic acid (HAA) |  |  |  |
| Continuous^a^ | 40.2 (31.0-52.7) | 1.00 (0.83,1.21) | 0.82 (0.66,1.03) |
| T1 | <36.4 | ref | ref |
| T2 | 26.4-51.9 | 0.90 (0.68,1.20) | 0.77 (0.57,1.05) |
| T3 | >37.4 | 1.11 (0.84,1.47) | 0.83 (0.61,1.12) |
| Picolinic acid (PA) |  |  |  |
| Continuous^a^ | 40.5 (28.6-55.9) | 0.87 (0.75,1.03) | **0.76 (0.64,0.92)** |
| T1 | <40.1 | ref | ref |
| T2 | 25.8-40.9 | 0.72 (0.54,0.95) | **0.65 (0.48,0.87)** |
| T3 | >37.7 | 0.85 (0.64,1.11) | **0.66 (0.49,0.88)** |
| Quinolinic acid (QA) |  |  |  |
| Continuous^a^ | 458.0 (361.0-603.0) | 1.88 (1.61,2.18) | **1.31 (1.05,1.63)** |
| T1 | <418.0 | ref | ref |
| T2 | 352.0-587.0 | 1.24 (0.90,1.71) | 0.98 (0.69,1.38) |
| T3 | >494.0 | 2.31 (1.73,3.09) | 1.32 (0.92,1.89) |
| ^1^Model adjusted using Cox proportional hazards regression for age, sex, tumor stage, tumor site, creatinine, and study cohort. Tertiles derived by study cohort. ^a^Analysis performed using log2-transformed concentrations. Thus, hazard ratios represent a doubling in concentrations. Trp, Kyn, PA, Kyn/Trp ratio: Total events: 290; total patients: 2100. QA, XA, KA: Total events: 290; total patients: 2101. AA, HK, HAA: Total events: 288; total patients: 2085. P-values <0.05 are statistically significant and are indicated in bold text. Abbreviations: Adj., adjusted; CI, confidence interval; HR, hazard ratio; IQR, interquartile range; T, tertile. | | | |

| **Table S4.** Associations between tryptophan-kynurenine pathway metabolites and all-cause mortality among colorectal cancer patients stratified by tumor site (ascending colon, descending colon, rectosigmoid junction/rectum).^1^ | | | | | | | | | | |
| --- | --- | --- | --- | --- | --- | --- | --- | --- | --- | --- |
|  | **Ascending colon** | |  | **Descending colon** | |  | **Rectosigmoid junction/rectum** | | |  |
|  | No. of deaths/ | Adj. HR |  | No. of events/ total No. of patients | Adj. HR |  | No. of events/ total No. of patients | Adj. HR |  |  |
|  | total No. of patients | (95% CI) | *P-value* |  | (95% CI) | *P-value* |  | (95% CI) | *P-value* | ***P_interaction_*** |
| ***All-cause mortality*** | | | | | | | | |  |  |
| **Tryptophan** |  |  |  |  |  |  |  |  |  |  |
| *(nmol/l)* |  |  |  |  |  |  |  |  |  |  |
| Continuous^a^ | 93/588 | **0.51 (0.30,0.87)** | 0.01 | 82/644 | 0.61 (0.31,1.21) | 0.16 | 94/757 | **0.44 (0.24,0.80)** | 0.007 | 0.05 |
| **Kynurenine** |  |  |  |  |  |  |  |  |  |  |
| *(nmol/l)* |  |  |  |  |  |  |  |  |  |  |
| Continuous^a^ | 93/588 | 1.67 (0.83,3.36) | 0.15 | 82/644 | 0.97 (0.46,2.06) | 0.94 | 94/757 | 1.12 (0.57,2.18) | 0.75 | 0.16 |
| **Kyn/Trp ratio** |  |  |  |  |  |  |  |  |  |  |
| *(Kyn/Trp)* |  |  |  |  |  |  |  |  |  |  |
| Continuous^a^ | 93/588 | **2.49 (1.53,4.04)** | 0.0002 | 82/644 | 1.55 (0.81,2.96) | 0.19 | 94/757 | **2.12 (1.24,3.63)** | 0.006 | 0.29 |
| **Kynurenic acid (KA)** |  |  |  |  |  |  |  |  |  |  |
| *(nmol/l)* |  |  |  |  |  |  |  |  |  |  |
| Continuous^a^ | 93/588 | 1.05 (0.69,1.59) | 0.82 | 82/644 | 0.67  (0.40,1.12) | 0.16 | 94/758 | 0.78 (0.51,1.20) | 0.25 | 0.32 |
| **Anthranilic acid (AA)** |  |  |  |  |  |  |  |  |  |  |
| *(nmol/l)* |  |  |  |  |  |  |  |  |  |  |
| Continuous^a^ | 92/585 | 0.94 (0.62,1.41) | 0.76 | 81/638 | 1.00 (0.63,1.61) | 0.99 | 94/754 | 0.83 (0.57,1.20) | 0.32 | 0.17 |
| **3-Hydroxykynurenine (HK)** |  |  |  |  |  |  |  |  |  |  |
| *(nmol/l)* |  |  |  |  |  |  |  |  |  |  |
| Continuous^a^ | 92/585 | **2.37 (1.69,3.34)** | <0.0001 | 81/638 | 1.42 (0.93,2.17) | 0.11 | 94/754 | **1.81 (1.30,2.53)** | 0.0005 | 0.96 |
| **Xanthurenic acid (XA)** |  |  |  |  |  |  |  |  |  |  |
| *(nmol/l)* |  |  |  |  |  |  |  |  |  |  |
| Continuous^a^ | 93/588 | 0.79 (0.62,1.00) | 0.05 | 82/644 | **0.67 (0.50,0.91)** | 0.009 | 94/758 | **0.71 (0.56,0.89)** | 0.003 | 0.39 |
| **3-Hydroxyanthranilic acid (HAA)** |  |  |  |  |  |  |  |  |  |  |
| *(nmol/l)* |  |  |  |  |  |  |  |  |  |  |
| Continuous^a^ | 92/585 | 0.96 (0.66,1.38) | 0.81 | 81/638 | 0.79 (0.51,1.22) | 0.29 | 94/754 | **0.68 (0.47,0.98)** | 0.04 | 0.12 |
| **Picolinic acid (PA)** |  |  |  |  |  |  |  |  |  |  |
| *(nmol/l)* |  |  |  |  |  |  |  |  |  |  |
| Continuous^a^ | 93/588 | 0.81 (0.60,1.09) | 0.16 | 82/643 | 0.80 (0.56,1.13) | 0.20 | 94/758 | **0.70 (0.51,0.95)** | 0.02 | 0.15 |
| **Quinolinic acid (QA)** |  |  |  |  |  |  |  |  |  |  |
| *(nmol/l)* |  |  |  |  |  |  |  |  |  |  |
| Continuous^a^ | 93/588 | **1.62 (1.16,2.28)** | 0.005 | 82/644 | 1.02 (0.62,1.68) | 0.95 | 94/758 | 1.13 (0.80,1.60) | 0.49 | **0.04** |
| ^1^Model adjusted using Cox proportional hazards regression for age, sex, tumor stage, creatinine, and study cohort. ^a^Analysis performed using log2-transformed concentrations. Thus, hazard ratios represent a doubling in concentrations. P-values <0.05 are statistically significant and are indicated in bold text. Abbreviations: Adj, adjusted; HR, hazard ratio; CI, confidence interval; Kyn/Trp ratio, kynurenine-to-tryptophan ratio; nmol, nanomolar; l, liter. | | | | | | | | | | |

| **Table S5.** Associations between tryptophan-kynurenine pathway metabolites and all-cause mortality among colorectal cancer patients stratified by tumor stage (stage I, II and III).^1^ | | | | | | | | | | | |
| --- | --- | --- | --- | --- | --- | --- | --- | --- | --- | --- | --- |
|  | **Stage I** | |  | **Stage II** | |  | **Stage III** | |  |  |  |
|  | No. of events/ | Adj. HR* |  | No. of events/ total No. of patients | Adj. HR* |  | No. of events/ total No. of patients | Adj. HR* |  |  |  |
|  | total No. of patients | (95% CI) | *P-value* |  | (95% CI) | *P-value* |  | (95% CI) | *P-value* | ***P_interaction_*** |  |
| ***All-cause mortality*** | |  |  |  |  |  |  |  |  |  |  |
| **Tryptophan** |  |  |  |  |  |  |  |  |  |  |  |
| *(nmol/l)* |  |  |  |  |  |  |  |  |  |  |  |
| Continuous^a^ | 48/552 | **0.30 (0.12, 0.72)** | 0.008 | 76/596 | **0.55 (0.31, 0.96)** | 0.04 | 145/841 | **0.62 (0.40, 0.98)** | 0.04 | 0.84 |  |
| **Kynurenine** |  |  |  |  |  |  |  |  |  |  |  |
| *(nmol/l)* |  |  |  |  |  |  |  |  |  |  |  |
| Continuous^a^ | 48/552 | **3.13 (1.15, 8.54)** | 0.03 | 76/596 | 1.66 (0.81, 3.41) | 0.17 | 145/841 | 0.81 (0.46, 1.45) | 0.48 | **<0.0001** |  |
| **Kyn/Trp ratio** |  |  |  |  |  |  |  |  |  |  |  |
| *(Kyn/Trp)* |  |  |  |  |  |  |  |  |  |  |  |
| Continuous^a^ | 48/552 | **6.78 (2.88, 16.0)** | <0.001 | 76/596 | **2.51 (1.44, 4.38)** | 0.001 | 145/841 | 1.39 (0.89, 2.17) | 0.15 | **<0.0001** |  |
| **Kynurenic acid (KA)** |  |  |  |  |  |  |  |  |  |  |  |
| *(nmol/l)* |  |  |  |  |  |  |  |  |  |  |  |
| Continuous^a^ | 48/553 | 1.04 (0.57, 1.91) | 0.90 | 76/596 | 0.93 (0.57, 1.53) | 0.78 | 145/841 | 0.72 (0.55, 1.08) | 0.13 | **0.004** |  |
| **Anthranilic acid (AA)** |  |  |  |  |  |  |  |  |  |  |  |
| *(nmol/l)* |  |  |  |  |  |  |  |  |  |  |  |
| Continuous^a^ | 48/553 | 1.00 (0.55, 1.81) | 0.99 | 75/594 | 0.96 (0.62, 1.49) | 0.86 | 144/838 | 0.81 (0.58, 1.13) | 0.21 | **0.03** |  |
| **3-Hydroxykynurenine (HK)** |  |  |  |  |  |  |  |  |  |  |  |
| *(nmol/l)* |  |  |  |  |  |  |  |  |  |  |  |
| Continuous^a^ | 48/545 | **3.53 (2.10, 5.93)** | <0.01 | 75/594 | **1.69 (1.18, 2.44)** | 0.005 | 144/838 | **1.62 (1.19, 2.20)** | 0.002 | **<0.0001** |  |
| **Xanthurenic acid (XA)** |  |  |  |  |  |  |  |  |  |  |  |
| *(nmol/l)* |  |  |  |  |  |  |  |  |  |  |  |
| Continuous^a^ | 48/553 | 0.71 (0.50, 1.02) | 0.06 | 76/596 | **0.72 (0.55, 0.94)** | 0.02 | 145/841 | **0.75 (0.62, 0.90)** | 0.003 | 0.17 |  |
| **3-Hydroxyanthranilic acid (HAA)** |  |  |  |  |  |  |  |  |  |  |  |
| *(nmol/l)* |  |  |  |  |  |  |  |  |  |  |  |
| Continuous^a^ | 48/545 | 1.04 (0.59, 1.83) | 0.89 | 75/594 | 0.94 (0.63, 1.41) | 0.77 | 144/838 | **0.68 (0.50, 0.93)** | 0.02 | **0.02** |  |
| **Picolinic acid (PA)** |  |  |  |  |  |  |  |  |  |  |  |
| *(nmol/l)* |  |  |  |  |  |  |  |  |  |  |  |
| Continuous^a^ | 48/553 | 1.13 (0.71, 1.80) | 0.62 | 76/596 | **0.68 (0.48, 0.95)** | 0.03 | 145/840 | **0.72 (0.56, 0.92)** | 0.009 | **0.005** |  |
| **Quinolinic acid (QA)** |  |  |  |  |  |  |  |  |  |  |  |
| *(nmol/l)* |  |  |  |  |  |  |  |  |  |  |  |
| Continuous^a^ | 48/553 | **3.49 (1.90, 6.41)** | <0.001 | 76/596 | 1.42 (0.97, 2.08) | 0.07 | 145/841 | 0.94 (0.68, 1.29) | 0.68 | **<0.0001** |  |
| ^1^Model adjusted using Cox proportional hazards regression for age, sex, tumor site, creatinine, and study cohort. ^a^Analysis performed using log2-transformed concentrations. Thus, hazard ratios represent a doubling in concentrations. P-values <0.05 are statistically significant and are indicated in bold text. Abbreviations: Adj, adjusted; HR, hazard ratio; CI, confidence interval; Kyn/Trp ratio, kynurenine-to-tryptophan ratio; nmol, nanomolar; l, liter. | | | | | | | | | | |  |

| **Table S6.** Associations between tryptophan-kynurenine pathway metabolites with all-cause mortality among colorectal cancer patients stratified by BMI (normal weight, overweight, obese).^1^ | | | | | | | | | | | |
| --- | --- | --- | --- | --- | --- | --- | --- | --- | --- | --- | --- |
|  | **Normal weight (≥18.5 BMI < 25)** | | | **Overweight (≥25 BMI < 30)** | | | **Obese (≥30 BMI)** | | |  | |
|  | No. of deaths/ | Adj. HR* |  | No. of events/ total No. of patients | Adj. HR* |  | No. of events/ total No. of patients | Adj. HR* |  |  |  |
|  | total No. of patients | (95% CI) | *P-value* |  | (95% CI) | *P-value* |  | (95% CI) | *P-value* | ***P_interaction_*** |  |
| ***All-cause mortality*** | |  |  |  |  |  |  |  |  |  |  |
| **Tryptophan** |  |  |  |  |  |  |  |  |  |  |  |
| *(nmol/l)* |  |  |  |  |  |  |  |  |  |  |  |
| Continuous^a^ | 81/682 | **0.52 (0.32, 0.87)** | **0.013** | 130/856 | **0.44 (0.28, 0.71)** | **<0.001** | 59/422 | 1.03 (0.45, 2.36) | 0.94 | 0.11 |  |
| **Kynurenine** |  |  |  |  |  |  |  |  |  |  |  |
| *(nmol/l)* |  |  |  |  |  |  |  |  |  |  |  |
| Continuous^a^ | 81/682 | 0.99 (0.47, 2.08) | 0.98 | 130/856 | 1.00 (0.54, 1.85) | 0.99 | 59/423 | **2.64 (1.09, 6.39)** | **0.03** | 0.13 |  |
| **Kyn/Trp ratio** |  |  |  |  |  |  |  |  |  |  |  |
| *(Kyn/Trp)* |  |  |  |  |  |  |  |  |  |  |  |
| Continuous^a^ | 81/682 | **1.98 (1.16, 3.38)** | **0.01** | 130/856 | **2.27 (1.42, 3.62)** | **<0.001** | 59/422 | 1.95 (0.92, 4.11) | 0.08 | 0.72 |  |
| **Kynurenic acid (KA)** |  |  |  |  |  |  |  |  |  |  |  |
| *(nmol/l)* |  |  |  |  |  |  |  |  |  |  |  |
| Continuous^a^ | 81/682 | **0.61 (0.39, 0.96)** | **0.03** | 130/856 | 0.97 (0.66, 1.42) | 0.87 | 59/423 | 0.85 (0.51, 1.41) | 0.53 | 0.06 |  |
| **Anthranilic acid (AA)** |  |  |  |  |  |  |  |  |  |  |  |
| *(nmol/l)* |  |  |  |  |  |  |  |  |  |  |  |
| Continuous^a^ | 81/682 | 1.13 (0.75, 1.71) | 0.55 | 129/852 | 0.83 (0.58, 1.17) | 0.28 | 59/423 | 1.38 (0.85, 2.23) | 0.19 | 0.09 |  |
| **3-Hydroxy-kynurenine (HK)** |  |  |  |  |  |  |  |  |  |  |  |
| *(nmol/l)* |  |  |  |  |  |  |  |  |  |  |  |
| Continuous^a^ | 81/676 | 1.40 (0.96, 2.04) | 0.08 | 129/852 | **1.73 (1.27, 2.35)** | **0.0005** | 59/423 | **2.23 (1.43, 3.49)** | **0.0004** | 0.19 |  |
| **Xanthurenic acid (XA)** |  |  |  |  |  |  |  |  |  |  |  |
| *(nmol/l)* |  |  |  |  |  |  |  |  |  |  |  |
| Continuous^a^ | 81/682 | **0.72 (0.57, 0.90)** | **0.004** | 130/856 | **0.78 (0.63, 0.97)** | **0.02** | 59/423 | **0.64 (0.47, 0.86)** | **0.003** | 0.15 |  |
| **3-Hydroxy-anthranilic acid (HAA)** |  |  |  |  |  |  |  |  |  |  |  |
| *(nmol/l)* |  |  |  |  |  |  |  |  |  |  |  |
| Continuous^a^ | 81/676 | 0.92 (0.63, 1.34) | 0.66 | 129/852 | **0.67 (0.48, 0.92)** | **0.01** | 58/420 | 0.89 (0.56, 1.43) | 0.63 | 0.28 |  |
| **Picolinic acid (PA)** |  |  |  |  |  |  |  |  |  |  |  |
| *(nmol/l)* |  |  |  |  |  |  |  |  |  |  |  |
| Continuous^a^ | 81/681 | 0.74 (0.54, 1.02) | 0.07 | 130/856 | **0.71 (0.55, 0.93)** | **0.01** | 59/423 | 0.86 (0.58, 1.29) | 0.46 | 0.37 |  |
| **Quinolinic acid (QA)** |  |  |  |  |  |  |  |  |  |  |  |
| *(nmol/l)* |  |  |  |  |  |  |  |  |  |  |  |
| Continuous^a^ | 81/682 | 1.28 (0.84, 1.94) | 0.25 | 130/856 | 1.26 (0.89, 1.78) | 0.18 | 59/423 | 1.44 (0.84, 2.47) | 0.19 | 0.38 |  |
| ^1^Model adjusted using Cox proportional hazards regression for age, sex, tumor site, creatinine, and study cohort. ^a^Analysis performed using log2-transformed concentrations. Thus, hazard ratios represent a doubling in concentrations. P-values <0.05 are statistically significant and are indicated in bold text. Abbreviations: Adj, adjusted; HR, hazard ratio; CI, confidence interval; Kyn/Trp ratio, kynurenine-to-tryptophan ratio; nmol, nanomolar; l, liter. | | | | | | | | | | |  |

| **Table S7.** Associations between tryptophan-kynurenine pathway metabolites and all-cause mortality among colorectal cancer patients stratified by study cohort.^1^ | | | | | |
| --- | --- | --- | --- | --- | --- |
|  | **Median (IQR)**  **concentration** |  | **All-cause mortality** | |  |
| **Biomarkers (nmol/l)**  **continuous^a^** |  |  | **No. of deaths/**  **total No. of patients** | **Adj. HR**  **(95% CI)** | ***P-value*** |
| **Tryptophan** | | | | |  |
| COLON | 61.81 (52.64,71.14) |  | 134/1060 | 0.30 (0.19,0.49) | **<0.0001** |
| EnCoRe | 62.28 (53.76,70.24) |  | 27/284 | 1.27 (0.38,4.3) | 0.70 |
| ColoCare FHCRC | 58.09 (52.07,73.09) |  | 24/130 | 0.81 (0.24,2.77) | 0.74 |
| ColoCare HCI | 62.70 (52.2,70.15) |  | 5/63 | 2.13 (0.02,193.0) | 0.74 |
| ColoCare HD | 58.78 (46.4,69.71) |  | 29/270 | 0.69 (0.32,1.46) | 0.33 |
| CORSA | 60.60 (52.94,69.05) |  | 50/182 | 0.54 (0.20,1.46) | 0.22 |
| **Kynurenine** |  |  |  |  |  |
| COLON | 1.63 (1.41,1.86) |  | 134/1060 | 0.60 (0.33,1.11) | 0.11 |
| EnCoRe | 1.67 (1.45,1.95) |  | 27/284 | 1.37 (0.38,4.97) | 0.63 |
| ColoCare FHCRC | 1.69 (1.48,1.98) |  | 24/130 | 2.66 (0.46,15.44) | 0.27 |
| ColoCare HCI | 1.68 (1.46,1.98) |  | 5/63 | 0.71 (0.03,20.02) | 0.84 |
| ColoCare HD | 1.62 (1.33,1.90) |  | 29/270 | 2.71 (0.82,8.98) | 0.10 |
| CORSA | 1.68 (1.40,1.95) |  | 50/182 | 3.00 (1.10,8.19) | **0.03** |
| **Kyn/Trp ratio** |  |  |  |  |  |
| COLON | 0.026 (0.022,0.031) |  | 134/1060 | 2.10 (1.34,3.29) | **0.001** |
| EnCoRe | 0.027 (0.023,0.031) |  | 27/284 | 1.04 (0.32,3.41) | 0.95 |
| ColoCare FHCRC | 0.028 (0.023,0.035) |  | 24/130 | 1.99 (0.58,6.81) | 0.27 |
| ColoCare HCI | 0.027 (0.024,0.033) |  | 5/63 | 0.30 (0.004,24.05) | 0.59 |
| ColoCare HD | 0.027 (0.022,0.035) |  | 29/270 | 3.05 (1.23,7.59) | **0.02** |
| CORSA | 0.027 (0.022,0.033) |  | 50/182 | 3.39 (1.50,7.63) | **0.003** |
| **Kynurenic acid** |  |  |  |  |  |
| COLON | 47.80 (27.80,62.10) |  | 134/1060 | 0.76 (0.52,1.10) | 0.15 |
| EnCoRe | 50.27 (40.40,64.30) |  | 27/284 | 0.60 (0.27,1.32) | 0.20 |
| ColoCare FHCRC | 44.00 (30.20,61.20) |  | 24/130 | 0.79 (0.34,1.85) | 0.59 |
| ColoCare HCI | 52.60 (36.20,71.70) |  | 5/63 | - | - |
| ColoCare HD | 44.20 (33.80,59.80) |  | 29/270 | 2.35 (1.04,5.32) | **0.04** |
| CORSA | 46.40 (35.70,60.60) |  | 50/182 | 0.88 (0.51,1.51) | 0.63 |
| **Anthranilic acid** |  |  |  |  |  |
| COLON | 15.00 (12.30,18.80) |  | 134/1059 | 0.78 (0.53,1.14) | 0.20 |
| EnCoRe | 16.20 (13.20,20.40) |  | 27/284 | 0.95 (0.38,2.35) | 0.90 |
| ColoCare FHCRC | 19.20 (13.47,26.60) |  | 23/127 | 1.04 (0.52,2.11) | 0.91 |
| ColoCare HCI | 25.00 (21.40,32.10) |  | 5/63 | 12.46 (0.36,426.35) | 0.16 |
| ColoCare HD | 23.50 (16.99,32.00) |  | 29/270 | 0.83 (0.47,1.49) | 0.53 |
| CORSA | 14.80 (11.50,20.40) |  | 49/174 | 0.96 (0.57,1.62) | 0.88 |
| **3-Hydroxykynurenine** |  |  |  |  |  |
| COLON | 44.50 (34.60,57.80) |  | 134/1059 | 1.54 (1.14,2.07) | **0.005** |
| EnCoRe | 42.50 (34.40,54.70) |  | 27/284 | 1.89 (0.90,3.95) | 0.09 |
| ColoCare FHCRC | 40.70 (30.50,54.30) |  | 23/127 | 1.95 (0.85,4.48) | 0.11 |
| ColoCare HCI | 46.40 (34.90,57.40) |  | 5/63 | 0.88 (0.09,8.50) | 0.91 |
| ColoCare HD | 47.70 (37.60,62.20) |  | 29/270 | 2.87 (1.58,5.23) | **0.0005** |
| CORSA | 52.90 (38.60,67.80) |  | 49/174 | 2.55 (1.50,4.35) | **0.0006** |
| **Xanthurenic acid** |  |  |  |  |  |
| COLON | 14.80 (9.98,20.20) |  | 134/1060 | 0.69 (0.56,0.85) | **0.0004** |
| EnCoRe | 14.80 (10.00,20.90) |  | 27/284 | 0.78 (0.47,1.29) | 0.34 |
| ColoCare FHCRC | 11.16 (6.62,17.40) |  | 24/130 | 0.77 (0.50,1.19) | 0.24 |
| ColoCare HCI | 12.02 (6.35,15.86) |  | 5/63 | 0.061 (0.004,0.94) | **0.04** |
| ColoCare HD | 10.34 (6.24,14.84) |  | 29/271 | 0.77 (0.52,1.13) | 0.18 |
| CORSA | 10.50 (7.09,15.00) |  | 50/182 | 0.79 (0.53,1.18) | 0.26 |
| **3-Hydroxyanthranilic acid** |  |  |  |  |  |
| COLON | 43.20 (33.50,56.10) |  | 134/1059 | 0.64 (0.46,0.89) | **0.007** |
| EnCoRe | 44.50 (33.70,56.70) |  | 27/284 | 1.04 (0.50,2.17) | 0.92 |
| ColoCare FHCRC | 33.20 (26.30,43.10) |  | 23/127 | 0.87 (0.41,1.81) | 0.41 |
| ColoCare HCI | 33.20 (24.30,46.40) |  | 5/63 | 0.23 (0.01,3.90) | 0.31 |
| ColoCare HD | 30.90 (22.40,41.230) |  | 29/270 | 0.94 (0.50,1.79) | 0.85 |
| CORSA | 38.30 (29.30,49.80) |  | 55/186 | 0.93 (0.54,1.62) | 0.81 |
| **Picolinic acid** |  |  |  |  |  |
| COLON | 42.40 (31.90,58.00) |  | 134/1060 | 0.62 (0.47,0.82) | **0.0007** |
| EnCoRe | 47.20 (34.90,62.60) |  | 27/284 | 0.84 (0.46,1.54) | 0.57 |
| ColoCare FHCRC | 39.40 (27.00,54.80) |  | 24/129 | 0.84 (0.48,1.46) | 0.53 |
| ColoCare HCI | 35.50 (24.60,54.20) |  | 5/63 | 0.95 (0.23,3.95) | 0.94 |
| ColoCare HD | 33.80 (22.00,44.40) |  | 29/271 | 0.80 (0.49,1.31) | 0.38 |
| CORSA | 31.20 (23.00,42.10) |  | 50/182 | 1.04 (0.66,1.64) | 0.86 |
| **Quinolinic acid** | | | | |  |
| COLON | 462.0 (369.0,594.0) |  | 135/1060 | 1.15 (0.80,1.67) | 0.44 |
| EnCoRe | 470.0 (375.0,618.0) |  | 27/284 | 1.40 (0.66,2.98) | 0.38 |
| ColoCare FHCRC | 408.0 (336.0,668.0) |  | 24/130 | 1.94 (0.83,4.54) | 0.12 |
| ColoCare HCI | 516.0 (378.0,645.0) |  | 5/63 | 10.01 (0.39,254.57) | 0.16 |
| ColoCare HD | 412.0 (294.0,576.0) |  | 29/271 | 1.69 (0.89,3.24) | 0.11 |
| CORSA | 468.0 (337.5,670.0) |  | 50/182 | 0.93 (0.58,1.48) | 0.74 |
| ^1^Model adjusted using Cox proportional hazards regression for age, sex, tumor stage, tumor site, and creatinine.  ^a^Analysis performed using log2-transformed concentrations. Thus, hazard ratios represent a doubling in concentrations. P-values <0.05 are statistically significant and are indicated in bold text. Abbreviations: Adj., adjusted; HR, hazard ratio; CI, confidence interval; nmol, Kyn/Trp ratio, kynurenine-to-tryptophan ratio; nanomolar; l, liter. | | | | | |

| **Table S8.** Associations between tryptophan-kynurenine pathway metabolites and all-cause mortality among colorectal cancer patients with at least 60 days follow-up time prior to an event.^1^ | | | | | |
| --- | --- | --- | --- | --- | --- |
|  |  | **All-cause mortality (All patients)** | | **All-cause mortality (patients with at least 60 days time prior to event)** | |
|  |  | No. of deaths/ | Adj. HR | No. of deaths/ | Adj. HR |
|  |  | total No. of patients | (95% CI) | total No. of patients | (95% CI) |
| **Tryptophan**  *(nmol/l)* | |  |  |  |  |
| Continuous^a^ |  | 290/ 2088 | 0.56 (0.41,0.77) | 252/ 1963 | 0.55 (0.39,0.77) |
| **Kynurenine**  *(nmol/l)* | |  |  |  |  |
| Continuous^a^ |  | 290/ 2088 | 1.28 (0.86,1.92) | 252/ 1963 | 1.31 (0.86,2.00) |
| **Kyn/Trp ratio**  *(Kyn/Trp)* |  |  |  |  |  |
| Continuous^a^ |  | 290/ 2088 | 2.07 (1.52,2.83) | 252/ 1963 | 2.11 (1.53,2.91) |
| **Kynurenic acid (KA)**  *(nmol/l)* | |  |  |  |  |
| Continuous^a^ |  | 290/ 2089 | 0.87 (0.68,1.12) | 252/ 1964 | 0.84 (0.65,1.09) |
| **Anthranilic acid (AA)**  *(nmol/l)* | |  |  |  |  |
| Continuous^a^ |  | 288/ 2073 | 0.92 (0.72,1.17) | 250/ 1951 | 0.93 (0.72,1.19) |
| **3-Hydroxykynurenine (HK)**  *(nmol/l)* | |  |  |  |  |
| Continuous^a^ |  | 288/ 2073 | 1.80 (1.47,2.21) | 250/ 1951 | 1.81 (1.46,2.24) |
| **Xanthurenic acid (XA)**  *(nmol/l)* | |  |  |  |  |
| Continuous^a^ |  | 290/ 2089 | 0.74 (0.64,0.85) | 252/ 1964 | 0.73 (0.63,0.84) |
| **3-Hydroxyanthranilic acid (HAA)**  *(nmol/l)* | |  |  |  |  |
| Continuous^a^ |  | 288/ 2073 | 0.82 (0.66,1.03) | 250/ 1951 | 0.83 (0.66,1.04) |
| **Picolinic acid (PA)**  *(nmol/l)* | |  |  |  |  |
| Continuous^a^ |  | 290/ 2088 | 0.76 (0.64,0.92) | 252/ 1963 | 0.75 (0.62,0.90) |
| **Quinolinic acid (QA)**  *(nmol/l)* | |  |  |  |  |
| Continuous^a^ |  | 290/ 2089 | 1.31 (1.05,1.63) | 252/ 1964 | 1.33 (1.06,1.67) |
| ^1^Model adjusted using Cox proportional hazards regression for age, sex, tumor stage and site, creatinine, and study cohort. ^a^Analysis performed using log2-transformed concentrations. Thus, hazard ratios represent a doubling in concentrations. Abbreviations: Adj., adjusted; HR, hazard ratio; CI, confidence interval; Kyn/Trp ratio, kynurenine-to-tryptophan ratio; nmol, nanomolar; l, liter. | | | | | |

| **Table S9.** Associations between tryptophan-kynurenine pathway metabolites and all-cause mortality among patients with stage I-III colorectal cancer additionally adjusted for BMI, smoking, or adherence to physical activity guidelines*: FOCUS Consortium.^1^ | | | | | | |
| --- | --- | --- | --- | --- | --- | --- |
|  | **All-cause mortality (BMI adj.)** | | **All-cause mortality (Smoking adj.)** | | **All-cause mortality (Physical activity adj.)** | |
|  | No. of deaths/ | Adj. HR* | No. of deaths/ | Adj. HR* | No. of deaths/ | Adj. HR* |
|  | total No. of patients | (95% CI) | total No. of patients | (95% CI) | total No. of patients | (95% CI) |
| **Tryptophan**  *(nmol/l)* |  |  |  |  |  |  |
| Continuous^a^ | 275/ 1978 | **0.57 (0.41,0.78)** | 275/ 1978 | **0.56 (0.41,0.77)** | 268/ 1968 | **0.56 (0.41,0.77)** |
| **Kynurenine**  *(nmol/l)* |  |  |  |  |  |  |
| Continuous^a^ | 275/ 1978 | 1.34 (0.90,2.01) | 275/ 1978 | 1.34 (0.90,2.01) | 268/ 1968 | 1.38 (0.92,2.07) |
| **Kyn/Trp ratio**  *(Kyn/Trp)* |  |  |  |  |  |  |
| Continuous^a^ | 275/ 1978 | **2.11 (1.55,2.87)** | 275/ 1978 | **2.11 (1.55,2.87)** | 268/1971 | **2.18 (1.60,2.96)** |
| **Kynurenic acid (KA)**  *(nmol/l)* |  |  |  |  |  |  |
| Continuous | 273/1966 | 0.90 (0.70,1.16) | 273/1966 | 0.90 (0.70,1.16) | 268/ 1969 | 0.91 (0.70,1.17) |
| **Anthranilic acid (AA)**  *(nmol/l)* |  |  |  |  |  |  |
| Continuous^a^ | 273/1982 | 0.90 (0.70,1.14) | 273/1982 | 0.90 (0.70,1.14) | 266/1956 | 0.87 (0.70,1.12) |
| **3-Hydroxykynurenine (HK)**  *(nmol/l)* |  |  |  |  |  |  |
| Continuous^a^ | 273/ 1966 | **1.77 (1.45,2.17)** | 273/ 1966 | **1.77 (1.45,2.17)** | 266/1956 | **1.79 (1.46,2.19)** |
| **Xanthurenic acid (XA)**  *(nmol/l)* |  |  |  |  |  |  |
| Continuous^a^ | 273/ 1979 | **0.75 (0.65,0.87)** | 273/ 1979 | **0.75 (0.65,0.87)** | 268/1969 | **0.74 (0.64,0.86)** |
| **3-Hydroxyanthranilic acid (HAA)**  *(nmol/l)* |  |  |  |  |  |  |
| Continuous^a^ | 273/ 1966 | 0.85 (0.68,1.06) | 273/ 1966 | 0.85 (0.68,1.06) | 266/1956 | 0.87 (0.70,1.08) |
| **Picolinic acid (PA)**  *(nmol/l)* |  |  |  |  |  |  |
| Continuous^a^ | 275/ 1978 | **0.78 (0.65,0.94)** | 275/ 1978 | **0.78 (0.65,0.94)** | 268/1969 | **0.76 (0.63,0.92)** |
| **Quinolinic acid (QA)**  *(nmol/l)* |  |  |  |  |  |  |
| Continuous | 275/ 1979 | **1.41 (1.12,1.77)** | 275/ 1979 | **1.41 (1.12,1.77)** | 268/1969 | **1.33 (1.07,1.66)** |
| ^1^Model adjusted using Cox proportional hazards regression for age, sex, tumor stage and site, creatinine, study cohort, and BMI (left panel), smoking (central panel) or adherence to physical activity guidelines (right panel).*Self-reported engagement in at least 150 min per week of moderate-to-vigorous physical activity. ^a^Analysis performed using log2-transformed concentrations. Thus, hazard ratios represent a doubling in concentrations. Abbreviations: Adj., adjusted; HR, hazard ratio; CI, confidence interval; Kyn/Trp ratio, kynurenine-to-tryptophan ratio; nmol, nanomolar; l, liter. | | | | | | |

| **Table S10.** Associations between tryptophan-kynurenine pathway metabolites and all-cause mortality among patients with stage I-III colorectal cancer additionally adjusted for neoadjuvant or adjuvant treatment: FOCUS Consortium.^1^ | | | | |
| --- | --- | --- | --- | --- |
|  | **All-cause mortality (Neoadjuvant adj.)** | | **All-cause mortality (Adjuvant adj.)** | |
|  | No. of deaths/ | Adj. HR* | No. of deaths/ | Adj. HR* |
|  | total No. of patients | (95% CI) | total No. of patients | (95% CI) |
| **Tryptophan**  *(nmol/l)* |  |  |  |  |
| Continuous^a^ | 275/ 1994 | **0.56 (0.41,0.76)** | 269/ 1912 | **0.53 (0.40,0.73)** |
| **Kynurenine**  *(nmol/l)* |  |  |  |  |
| Continuous^a^ | 275/ 1995 | 1.33 (0.89,1.98) | 269/1912 | 1.22 (0.81,1.83) |
| **Kyn/Trp ratio**  *(Kyn/Trp)* |  |  |  |  |
| Continuous^a^ | 275/ 1994 | **2.11 (1.56,2.86)** | 269/ 1912 | **2.10 (1.54,2.86)** |
| **Kynurenic acid (KA)**  *(nmol/l)* |  |  |  |  |
| Continuous^a^ | 269/1911 | 0.88 (0.68,1.13) | 269/1913 | 0.87 (0.68,1.13) |
| **Anthralinic acid (AA)**  *(nmol/l)* |  |  |  |  |
| Continuous^a^ | 273/1982 | 0.90 (0.71,1.14) | 267/1900 | 0.89 (0.70,1.13) |
| **3-Hydroxykynurenine (HK)**  *(nmol/l)* |  |  |  |  |
| Continuous^a^ | 273/ 1982 | **1.78 (1.46,2.18)** | 267/ 1900 | **1.76 (1.43,2.16)** |
| **Xanthurenic acid (XA)**  *(nmol/l)* |  |  |  |  |
| Continuous^a^ | 275/ 1995 | **0.73 (0.64,0.85)** | 269/ 1913 | **0.73 (0.63,0.85)** |
| **3-Hydroxyanthranilic acid (HAA)**  *(nmol/l)* |  |  |  |  |
| Continuous^a^ | 273/ 1982 | 0.83 (0.67,1.03) | 267/ 1900 | 0.82 (0.66,1.02) |
| **Picolinic acid (PA)**  *(nmol/l)* |  |  |  |  |
| Continuous^a^ | 275/ 1994 | **0.76 (0.64,0.92)** | 269/ 1912 | **0.77 (0.64,0.92)** |
| **Quinolinic acid (QA)**  *(nmol/l)* |  |  |  |  |
| Continuous^a^ | 275/ 1995 | **1.34 (1.08,1.66)** | 269/ 1913 | **1.31 (1.05,1.63)** |
| ^1^Model adjusted using Cox proportional hazards regression for age, sex, tumor stage and site, creatinine, study cohort, and neoadjuvant (left panel) or adjuvant treatment (right panel). ^a^Analysis performed using log2-transformed concentrations. Thus, hazard ratios represent a doubling in concentrations. Abbreviations: Adj., adjusted; HR, hazard ratio; CI, confidence interval; Kyn/Trp ratio, kynurenine-to-tryptophan ratio; nmol, nanomolar; l, liter. | | | | |
